# Cleavage of osmosensitive transcriptional factor NFAT5 by Coxsackieviral protease 2A promotes viral replication

**DOI:** 10.1371/journal.ppat.1006744

**Published:** 2017-12-08

**Authors:** Ye Qiu, Xin Ye, Huifang Mary Zhang, Paul Hanson, Guangze Zhao, Lei Tong, Ronald Xie, Decheng Yang

**Affiliations:** 1 Department of Pathology and Laboratory Medicine, University of British Columbia, Vancouver, Canada; 2 The Centre for Heart Lung Innovation, St. Paul’s Hospital, Vancouver, Canada; San Diego State University, UNITED STATES

## Abstract

Nuclear factor of activated T cells 5 (NFAT5)/Tonicity enhancer binding protein (TonEBP) is a transcription factor induced by hypertonic stress in the kidney. However, the function of NFAT5 in other organs has rarely been studied, even though it is ubiquitously expressed. Indeed, although NFAT5 was reported to be critical for heart development and function, its role in infectious heart diseases has remained obscure. In this study, we aimed to understand the mechanism by which NFAT5 interferes with infection of Coxsackievirus B3 (CVB3), a major cause of viral myocarditis. Our initial results demonstrated that although the mRNA level of NFAT5 remained constant during CVB3 infection, NFAT5 protein level decreased because the protein was cleaved. Bioinformatic prediction and verification of the predicted site by site-directed mutagenesis experiments determined that the NFAT5 protein was cleaved by CVB3 protease 2A at Glycine 503. Such cleavage led to the inactivation of NFAT5, and the 70-kDa N-terminal cleavage product (p70-NFAT5) exerted a dominant negative effect on the full-length NFAT5 protein. We further showed that elevated expression of NFAT5 to counteract viral protease cleavage, especially overexpression of a non-cleavable mutant of NFAT5, significantly inhibited CVB3 replication. Ectopic expression of NFAT5 resulted in elevated expression of inducible nitric oxide synthase (iNOS), a factor reported to inhibit CVB3 replication. The necessity of iNOS for the anti-CVB3 effect of NFAT5 was supported by the observation that inhibition of iNOS blocked the anti-CVB3 effect of NFAT5. In a murine model of viral myocarditis, we observed that treatment with hypertonic saline or mannitol solution upregulated NFAT5 and iNOS expression, inhibited CVB3 replication and reduced tissue damage in the heart. Taken together, our data demonstrate that the anti-CVB3 activity of NFAT5 is impaired during CVB3 infection due to 2A-mediated cleavage of NFAT5. Thus induction of NFAT5 by hypertonic agents may be a promising strategy for the development of anti-CVB3 therapeutics.

## Author summary

Coxsackievirus B3 (CVB3) is one of the predominant pathogens of viral myocarditis, which is a major cause of sudden death in children and young adults. CVB3 alters the expression of many proteins in host cells to facilitate its multiplication. Nuclear factor of activated T cells 5 (NFAT5) is known to be involved in the response to high salt concentration in body fluids but its role in viral infection is not currently understood. Here, we showed that NFAT5 was cleaved into several fragments during CVB3 infection, which blocked the activity of the protein. We demonstrated that intact NFAT5 inhibited CVB3 multiplication, but that such antiviral activity was impaired by NFAT5 cleavage products, indicating that CVB3 cleaves the NFAT5 protein as a survival strategy. When we used a high concentration of saline or mannitol solution to induce NFAT5 production in CVB3-infected cells and mice, we found that viral multiplication was significantly reduced, suggesting that there is therapeutic potential for use of NFAT5 to combat CVB3 infection. Taken together, our findings have uncovered a novel anti-CVB3 role of NFAT5 and provided a promising drug target for CVB3-induced myocarditis.

## Introduction

Viral myocarditis is an inflammatory heart disease caused by viral infection. Acute viral myocarditis can be lethal in a short period of time and chronic viral myocarditis frequently progresses to dilated cardiomyopathy (DCM), a severe heart disease for which the only current treatment is heart transplantation [1–3]. In patients less than 40 years of age, viral myocarditis and DCM are the major natural causes of sudden unexpected death, accounting for as many as 20% of all sudden death cases [4–6]. There are no effective drugs available for the treatment of viral myocarditis, partly due to the sporadic occurrence and rapid development of this disease.

In this study, we aimed to understand the molecular pathogenesis of viral myocarditis and hopefully to discover new strategies for treatment. CVB3 is a member of the enterovirus genus in the *Picornaviridae* family. The genome of CVB3 is a positive, single-stranded RNA molecule encoding a single open reading frame. The viral genome transcription depends on its own RNA-dependent RNA polymerase 3D. The genome also contains an internal ribosome entry site (IRES) within the 5’ untranslated region. Upon infection, the viral RNA is translated by a cap-independent/IRES-driven mechanism into a long polyprotein, which is processed by viral proteases 2A and 3C [7, 8]. In addition to processing viral polyproteins to complete the viral life cycle, CVB3 proteases also cleave various host proteins, modulating cellular signaling pathways to benefit viral replication. Several well-documented examples of such cleavage are the cleavage of eukaryotic translation initiation factor 4G (eIF4G) by protease 2A, shutting down host cap-dependent translation [9, 10], as well as cleavage of poly-A binding protein (PABP), inhibiting host translation initiation [11], and cleavage of cardiac dystrophin, a structural protein required for maintenance of cardiomyocyte architecture, which results in structural damage [12]. In addition, both 2A and 3C cleave Bid, a Bax-like BH3 protein, inducing the intrinsic mitochondria-mediated apoptosis pathway and leading to cell death [10].

Nuclear factor of activated T cells 5 (NFAT5)/Tonicity enhancer binding protein (TonEBP) is a master transcription factor (~170 kDa) activated in hypertonic stress. NFAT5 activation promotes cell survival in hypertonic conditions via stimulation of the expression of different tonicity-responsive genes by binding to the tonicity-responsive enhancer (TonE) located in these genes’ promoters [13]. Most NFAT5 studies have been conducted in the kidney, as this is the most common hypertonic environment in mammals. However, NFAT5 is actually ubiquitously expressed in almost all organs including those not exposed to hypertonic environments, such as the brain, heart, and skeletal muscles [14, 15], but the function of the protein in these organs has rarely been studied. Recently, a few reports have suggested the possible protective role of NFAT5 in some cases of heart injury. For instance, degradation of NFAT5 is induced in cardiomyocytes treated with doxorubicin, a commonly used anti-tumor agent, and a concurrent decrease of NFAT5 exacerbates myocyte death upon doxorubicin treatment [16]. In certain clinical trials, hypertonic saline solution (HSS), a common agent for inducing hypertonic stress, was used as a auxiliary supplement to improve cardiac function in heart diseases [17, 18]. The protective role of NFAT5 may be associated with the transcriptional activity of nuclear factor κB (NFκB). NFAT5 enhances NFκB activity by forming an NFκB-NFAT5 complex and enhancing NFκB’s binding to the κB elements of NFκB–responsive genes [19]. NFκB has been shown to influence numerous cardiovascular diseases and can protect cardiovascular tissues from injury [20]. More interestingly, NFκB has also been reported to promote host cell viability in CVB3 infection [21] and inducible nitric oxide synthase (iNOS), a downstream signal of NFκB, exerts inhibitory effects on CVB3 replication and decreases mouse mortality rate after viral infection [22]. Combined, these previous findings inspired us to link NFAT5 to anti-CVB3 activity, a virgin field for studies of both NFAT5 and viral myocarditis.

In this study, we found that the reduction of NFAT5 expression during CVB3 infection is primarily due to the post-translational cleavage of NFAT5 at glycine (G) 503 by CVB3 protease. Such cleavage impairs NFAT5 activity due to the dominant negative effect of the ~70 kDa N-terminal cleavage product (p70-NFAT5). Ectopic expression of the full-length (FL) NFAT5, especially a non-cleavable mutant of NFAT5, resulted in strong inhibition of CVB3 replication, suggesting an antiviral effect of NFAT5. These data were further substantiated by siRNA-mediated gene silencing of NFAT5, showing an increase of CVB3 replication. Mechanistic analyses revealed that iNOS, a downstream protein of NFAT5, is activated by full-length (FL) NFAT5 but inhibited by p70-NFAT5. Furthermore inhibition of iNOS by a specific inhibitor (1400W) or iNOS-specific siRNA blocked the antiviral effect of NFAT5. In treatment studies using cell cultures, we found that HSS or mannitol, two potential inducers of NFAT5, significantly inhibited CVB3 replication. Finally, using a CVB3 myocarditis mouse model, we demonstrated that treating the infected mice with HSS decreased viral load and alleviated heart tissue damage. In all, our studies uncovered a novel cardiac protective role of NFAT5 against CVB3 infection, revealing an underlying mechanism (activation of iNOS) by which NFAT5 protects the heart from CVB3 damage. Hypertonic agents may therefore have therapeutic potential against CVB3 infection and viral myocarditis.

## Results

### CVB3 infection decreases NFAT5 protein level but not mRNA level

**Fig 1:**
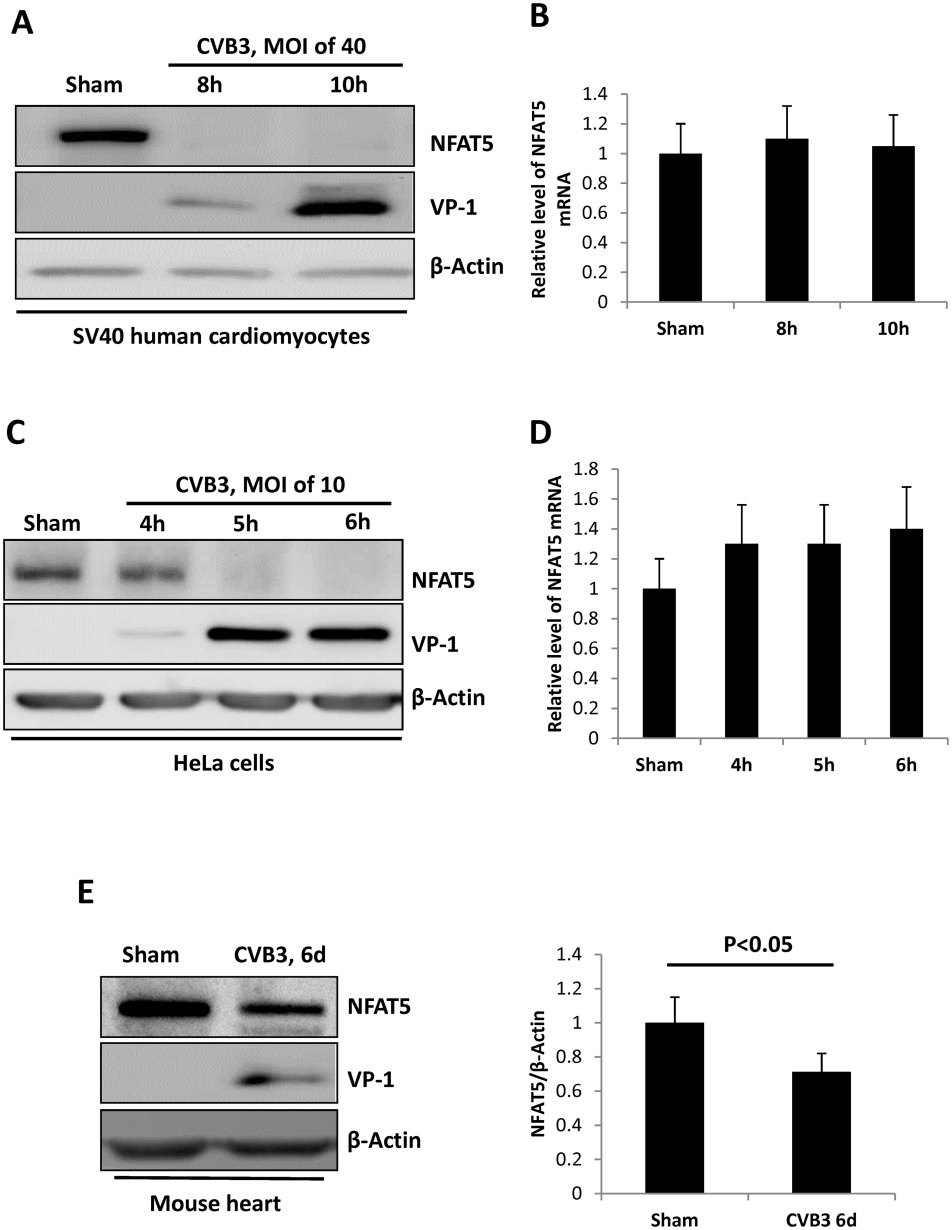
CVB3 infection reduces NFAT5 protein but not mRNA. SV40 human cardiomyocytes and HeLa cells were infected with CVB3 at an MOI of 40 or 10, respectively, or sham-infected with PBS and harvested at the indicated time points pi. Cellular proteins and RNAs were extracted for Western blot analysis of NFAT5 protein **(A, C)** and qPCR measurement of NFAT5 mRNA **(B, D)**, respectively. In qPCR assay, the result was shown as the relative level of each mRNA normalized to the level of GAPDH mRNA in the sample. Three biological replicates were performed for each assay. **(E)** 4-week-old A/J mice were infected with CVB3 at 10^5^ pfu (plaque forming unit) or sham-infected with PBS. At 6 days pi, the mice were sacrificed and the heart tissue was homogenized for Western blot analysis of NFAT5 protein. β-actin was used as a loading control. Quantitation of NFAT5 protein was conducted by densitometry analysis using the NIH ImageJ program (right panel). Three biological replicates were performed and the result was subjected to statistical analysis.

We first assessed the change of NFAT5 in SV40 human cardiomyocytes (HCM). HCM were infected with CVB3 at a multiplicity of infection (MOI) of 40 or sham-infected with phosphate-buffered saline (PBS) and then protein and RNA were extracted from the cells at 8 or 10 h post infection (pi). Western blot analyses using antibodies against CVB3 capsid protein (VP1) and NFAT5 were conducted to detect the level of CVB3 infection and CVB3-induced NFAT5 protein reduction, respectively. Fig 1A shows that NFAT5 protein was highly expressed in sham-infected HCM but was undetectable at 8 h pi; however, the mRNA levels detected by quantitative real-time PCR (qPCR) using NFAT5-specific primers remained unchanged (Fig 1B). Similar results were observed in HeLa cells infected with CVB3 at an MOI of 10, which showed a dramatic decrease of NFAT5 protein beginning at 5 h pi (Fig 1C), but no significant change in NFAT5 mRNA (Fig 1D). To test whether the observed changes in NFAT5 occurred *in vivo*, we infected 4-week-old A/J mice with 10^5^ plaque-forming unit (pfu) of CVB3 for six days and found that NFAT5 protein was decreased in the heart, the organ most susceptible to CVB3 infection (Fig 1E). Together, these results suggest that CVB3 infection mainly affects NFAT5 at the protein level.

### NFAT5 is cleaved by CVB3 proteases 2A and 3C

**Fig 2:**
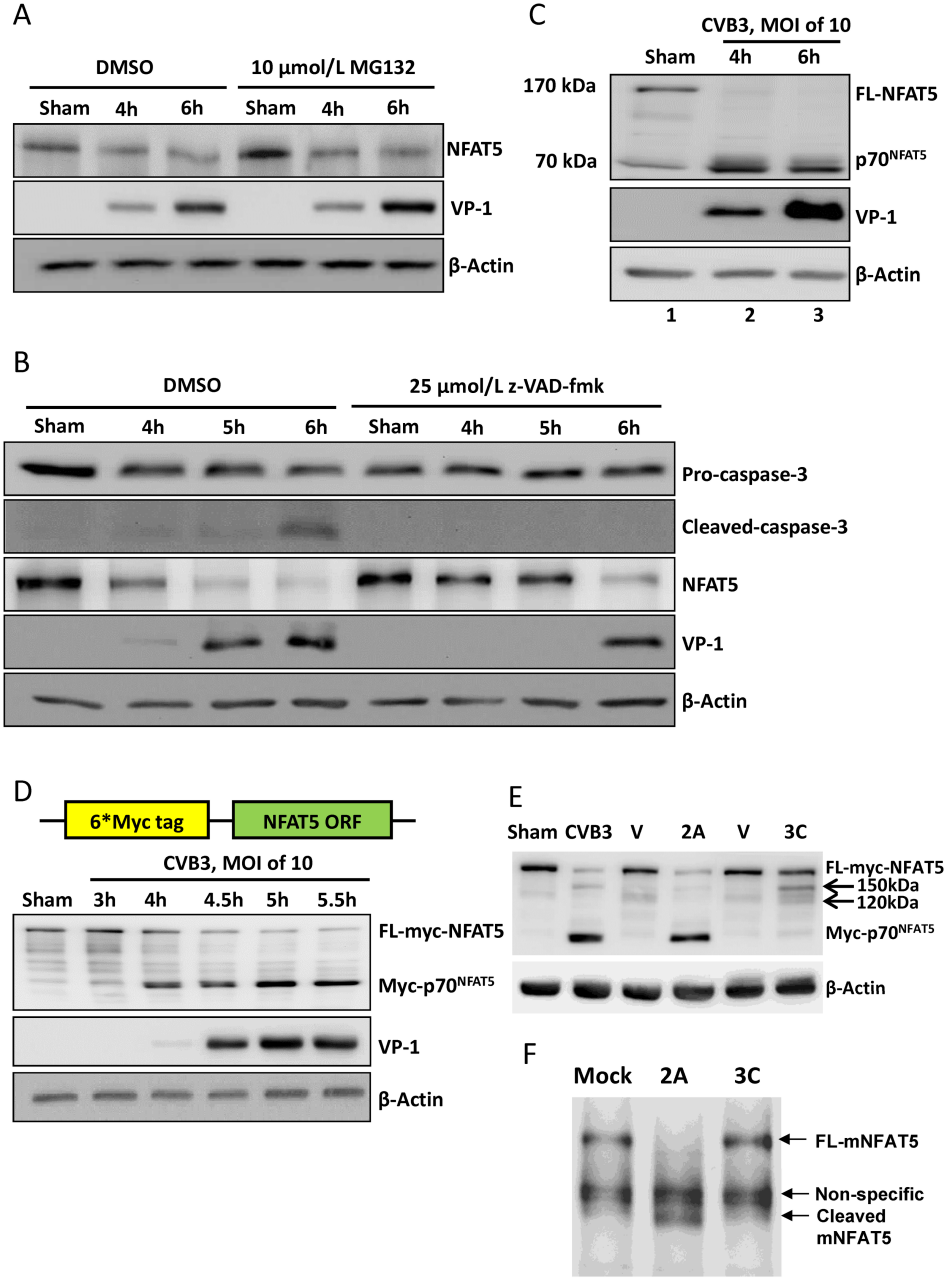
NFAT5 is cleaved by viral proteases 2A and 3C. HeLa cells were treated with 10 μM MG132 **(A)** or 25 μM z-VAD-fmk **(B)** and then infected with CVB3 at an MOI of 10. At indicated time points pi, the cellular proteins were subjected to Western blot analysis of NFAT5 and other proteins using the indicated antibodies. β-actin was used as a loading control. **(C)** HeLa cells were infected by CVB3 at an MOI of 10 for 4 and 6 h and then subjected to Western blot analysis using an antibody against the N-terminal epitope of NFAT5. **(D)** HeLa cells transfected with a plasmid expressing the 6*myc-NFAT5 fusion protein (upper panel) were infected with CVB3 or sham-infected as described above and subjected to Western blot analysis using an antibody against myc tag (lower panel). **(E)** HeLa cells expressing myc-NFAT5 were transfected with pIRES-2A (2A), pIRES-3C (3C) or vector only (V). At 36 h pt, the cells were subjected to Western blot analysis using an antibody against myc tag. The cells infected with CVB3 or sham-infected with PBS were used as controls. Arrows indicate the 3C cleavage bands. **(F)** Tissue homogenate from mouse heart was incubated with recombinant 2A or 3C for 8 h. Then the mouse NFAT5 (mNFAT5) N-terminal epitope was detected by Western blot using a specific antibody.

CVB3 infection promotes ubiquitin/proteasome-mediated protein degradation and induces caspase-3 activation [23, 24]. Since no change of NFAT5 mRNA was observed in CVB3 infection, we speculated that the decrease of NFAT5 protein might be due to proteasome-mediated degradation or caspase-3-mediated cleavage. To test this hypothesis, we used 10 μM MG-132, a proteasome inhibitor, and 50 μM z-VAD-fmk, a pan-caspase inhibitor, to treat CVB3-infected HeLa cells. To our surprise, neither MG-132 nor z-VAD-fmk was capable of blocking the decrease of NFAT5 protein (Fig 2A and 2B). These results indicate that NFAT5 decrease is not a result of proteasome-mediated degradation or caspase-3-mediated cleavage.

We then hypothesized that NFAT5 was possibly cleaved by CVB3 proteases 2A and/or 3C. To verify this hypothesis, we first detected the cleavage products of NFAT5 by Western blot analysis using an anti-NFAT5 N-terminal antibody and observed a ~70 kDa band appearing at 4 h pi, consistent with the reduction of 170 kDa FL NFAT5 (Fig 2C). However, a non-specific weaker band at ~70 kDa also appeared in sham-infected control (Fig 2C, **Lane 1**). To confirm the specificity of this cleavage, we constructed pEGFP-myc-NFAT5, a plasmid expressing NFAT5 tagged with a 6-myc peptide at its N-terminus, and transfected this plasmid into HeLa cells followed by infection with CVB3. By immunoblotting using an anti-myc antibody, we observed a ~76 kDa band at 4 h pi (Fig 2D), suggesting that the 70 kDa band was truly the N-terminal cleavage product of NFAT5 (p70-NFAT5) since the total molecular weight of the 6-myc tag is ~6 kDa. Then we attempted to determine which viral protease cleaved NFAT5 during CVB3 infection. For this aim, we transfected HeLa cells expressing myc-NFAT5 with plasmid pIRES-2A or pIRES-3C which expressed 2A or 3C, respectively. At 36 h post transfection (pt), by immunoblotting using a myc antibody, we observed that FL myc-NFAT5 decreased in both 2A- and 3C-transfected cells, but myc-p70-NFAT5 appeared only in 2A-transfected cells, which was similar to that in CVB3-infected cells, while 3C-transfected cells showed two weak bands of ~120 kDa and ~150 kDa (Fig 2E). To further confirm that such cleavage is not specific in human cancer cells such as HeLa cells, we performed the *in vitro* cleavage assay on mouse heart homogenate using recombinant 2A and 3C. We found that after the protease treatment for 8 h, mouse cardiac NFAT5 was cleaved by 2A but not 3C, as assessed by Western blot using the NFAT5 antibody mentioned above (Fig 2F), indicating that 2A-mediated cleavage of NFAT5 also occurred in mouse cardiac NFAT5. These results demonstrate that both 2A and 3C cleave NFAT5 but only 2A cleavage produces the p70-NFAT5 fragment.

### CVB3 protease 2A cleaves NFAT5 at G503

**Fig 3:**
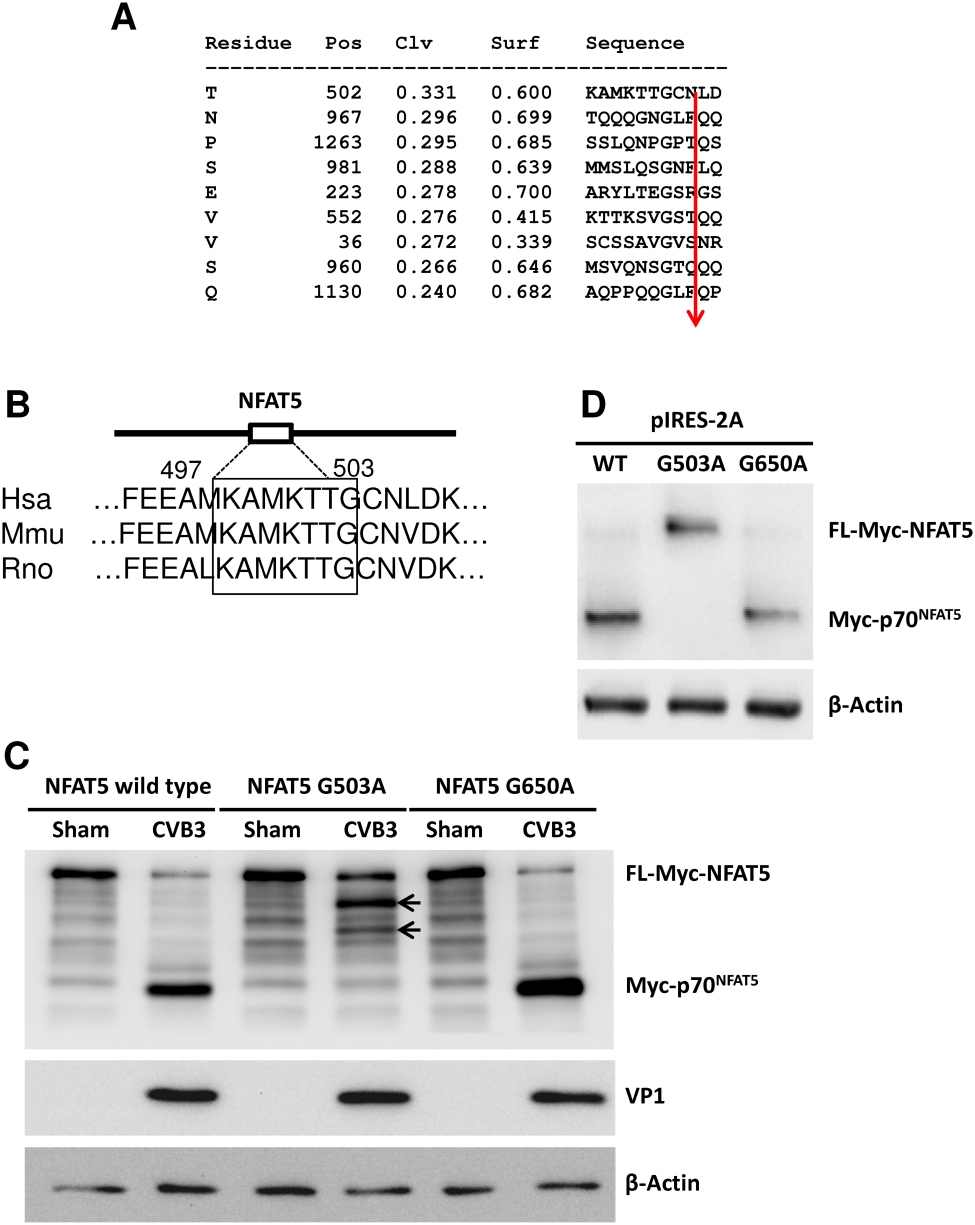
NFAT5 is cleaved by viral protease 2A at G503. **(A)** The potential 2A cleavage sites predicted by using the NetPicoRNA 1.0 program showing the cleavage position (Pos), cleavage score (Clv) and surface score (Surf). The arrow indicates the exact cleavage locus. **(B)** Alignment of the sequences around G503 of NFAT5 from different species. **(C)** HeLa cells were transfected with a plasmid expressing myc-NFAT5, myc-NFAT5^G503A^ or myc-NFAT5^G650A^ and then infected with CVB3 at an MOI of 10 for 6 h. Then the cell lysates were subjected to Western blot analysis using an anti-myc antibody. Arrows indicate the 3C cleavage bands. **(D)** HeLa cells were co-transfected with pIRES-2A and a plasmid expressing WT or mutant NFAT5^G503A^. At 36 h pt, the cell lysates were subjected to Western blot analysis using an anti-myc antibody.

According to the structural domains of NFAT5 protein, p70-NFAT5 contains most of the functional domains of NFAT5 (S1 Fig), including all domains necessary for nucleus-cytoplasm shuttling [25]. Interestingly, amino acid (aa) 175–471 within the N-terminal fragment of NFAT5 has been proven to function as a dominant negative mutant of NFAT5 [26, 27], implying the potential influence of p70-NFAT5 on overall NFAT5 activity. Therefore, we focused our study on p70-NFAT5, the 2A cleavage product. We utilized the program NetPicoRNA 1.0 [28] to analyze the whole aa sequence of NFAT5 and predicted the peptide bond just before G503 as the top candidate site of 2A cleavage (Fig 3A). Interestingly, the predicted 2A recognition motif on NFAT5 is conserved among different species including mice (Fig 3B), suggesting that both human and mouse NFAT5 could be cleaved by 2A. To verify the prediction, we mutated G503 of myc-NFAT5 to alanine (A) and constructed the expression plasmid pEGFP-myc-NFAT5^G503A^. We also made a second mutant plasmid pEGFP-myc-NFAT5^G650A^ as a control. We transfected the two mutants as well as wild-type (WT) NFAT5 into HeLa cells separately, and then infected the transfected cells with CVB3. Immunoblotting using an anti-myc antibody showed no myc-p70-NFAT5 band in NFAT5^G503A^ transfected cells, but instead a similar 3C cleavage pattern as WT NFAT5 (Fig 3C, **arrows**) as seen in Fig 2E, indicating that upon G503 mutation, 2A can no longer cleave NFAT5. As for the NFAT5^G650A^ control, we observed the same myc-p70-NFAT5 band as seen in WT NFAT5 cells after CVB infection (Fig 3C), suggesting that the mutagenesis procedure exerted no influence on NFAT5 cleavage. This result was further confirmed by 2A plasmid transfection, which showed no cleavage of FL myc-NFAT5^G503A^ upon 2A expression (Fig 3D). These data suggest that G503 is the only CVB3 protease 2A cleavage site on NFAT5.

### NFAT5 inhibits CVB3 replication

**Fig 4:**
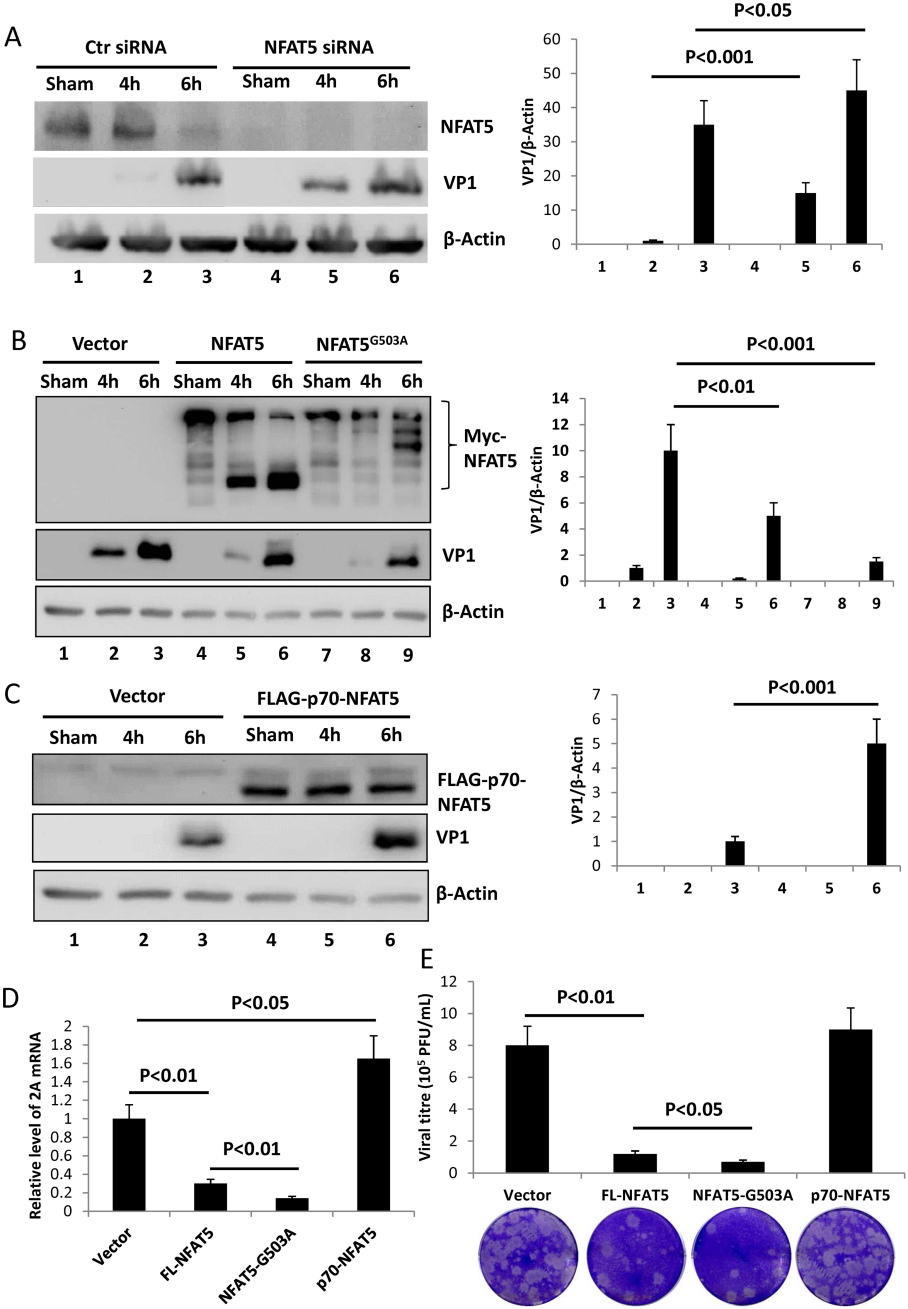
NFAT5 inhibits CVB3 replication while the N-terminal cleavage product p70-NFAT5 promotes CVB3 replication. **(A)** HeLa cells were transfected with NFAT5 siRNAs or scrambled control siRNAs (Ctr siRNA) and then infected with CVB3. At 4 and 6 h pi, the cell lysates were subjected to Western blot analysis of NFAT5 and VP1. Quantitation of VP1 was conducted by densitometry scan using the NIH ImageJ program and normalized against β-actin control (right panel). **(B)** HeLa cells were transfected with plasmids expressing WT myc-NFAT5 or myc-NFAT5^G503A^, infected with CVB3 and then harvested for VP1 detection and quantification as described above (right panel). **(C)** HeLa cells were transfected with plasmids expressing p70-NFAT5 tagged with FLAG and then infected with CVB3 for VP1 detection and quantification (right panel) at 6 h pi. **(D-E**) HeLa cells were transfected with plasmids expressing FL-NFAT5, NFAT5^G503A^ (NFAT5-G503A) or p70-NFAT5 and then infected with CVB3. At 6 h pi, the cellular RNAs were extracted for qPCR measurement of CVB3 genomic RNA level (normalized to GAPDH as described in Fig 1) **(D)** and the medium was collected for plaque assay to measure viral titre **(E**). Three biological replicates were performed for each assay and the result was subjected to statistical analysis.

Having confirmed the cleavage of NFAT5 by CVB3 protease 2A, our next focus was to study the effect of NFAT5 and NFAT5 cleavage on CVB3 replication. First, we knocked down NFAT5 expression using specific siRNAs in HeLa cells and then infected the cells with CVB3. At 4 and 6 h pi, we measured the level of VP1 to evaluate the viral replication in the cells. Immunoblotting results showed a 15-fold increase of VP1 in NFAT5-knockdown cells compared to control cells treated with scrambled siRNAs at 4 h pi. At 6 h pi, there was a statistically significant 20% increase in VP1 when NFAT5 was knocked down (Fig 4A). These results imply a potential anti-CVB3 activity of NFAT5. To further verify this speculation, we detected the level of VP1 in CVB3-infected cells overexpressing WT NFAT5, and observed a >50% decrease of VP1 in NFAT5-overexpressing cells compared to vector-transfected control cells (Fig 4B, **Lanes 2–3 vs. Lanes 5–6)**. More interestingly, in the cells overexpressing the non-cleavable NFAT5^G503A^, VP1 was decreased further (by ~80%) compared to vector-transfected control cells (Fig 4B, **Lanes 2–3 vs. Lanes 8–9**). This result may be explained by the speculation that FL NFAT5 plays a role in suppression of CVB3 replication and cleavage product p70-NFAT5 may play a dominant negative effect on NFAT5 activity. To verify this conjecture, we constructed a FLAG-tagged p70-NFAT5 plasmid to overexpress p70-NFAT5 in HeLa cells infected with CVB3. As expected, a ~4-fold increase of VP1 was observed in p70-NFAT5-overexpressing cells compared to vector-transfected control cells (Fig 4C). In addition to VP1 level, we also detected changes in CVB3 RNA and viral progeny release when FL NFAT5, NFAT5^G503A^ mutant or p70-NFAT5 were overexpressed. The qPCR results using primers flanking the coding region of CVB3 2A showed that FL NFAT5 decreased viral RNA level and that NFAT5^G503A^ further decreased it while p70-NFAT5 increased it (Fig 4D), which correlated well with the levels of VP1 shown in Fig 4C. For viral particle formation, plaque assay showed that FL NFAT5 and and NFAT5^G503A^ significantly decreased the viral particle release into the extracellular medium, while p70-NFAT5 had no effect on viral progeny release (Fig 4E). Taken together, these results suggest that FL NFAT5 inhibits viral replication, and that the N-terminal cleavage product p70-NFAT5 is capable of promoting the synthesis of viral protein and RNA.

### The 2A cleavage product p70-NFAT5 shows a dominant negative effect on NFAT5

**Fig 5:**
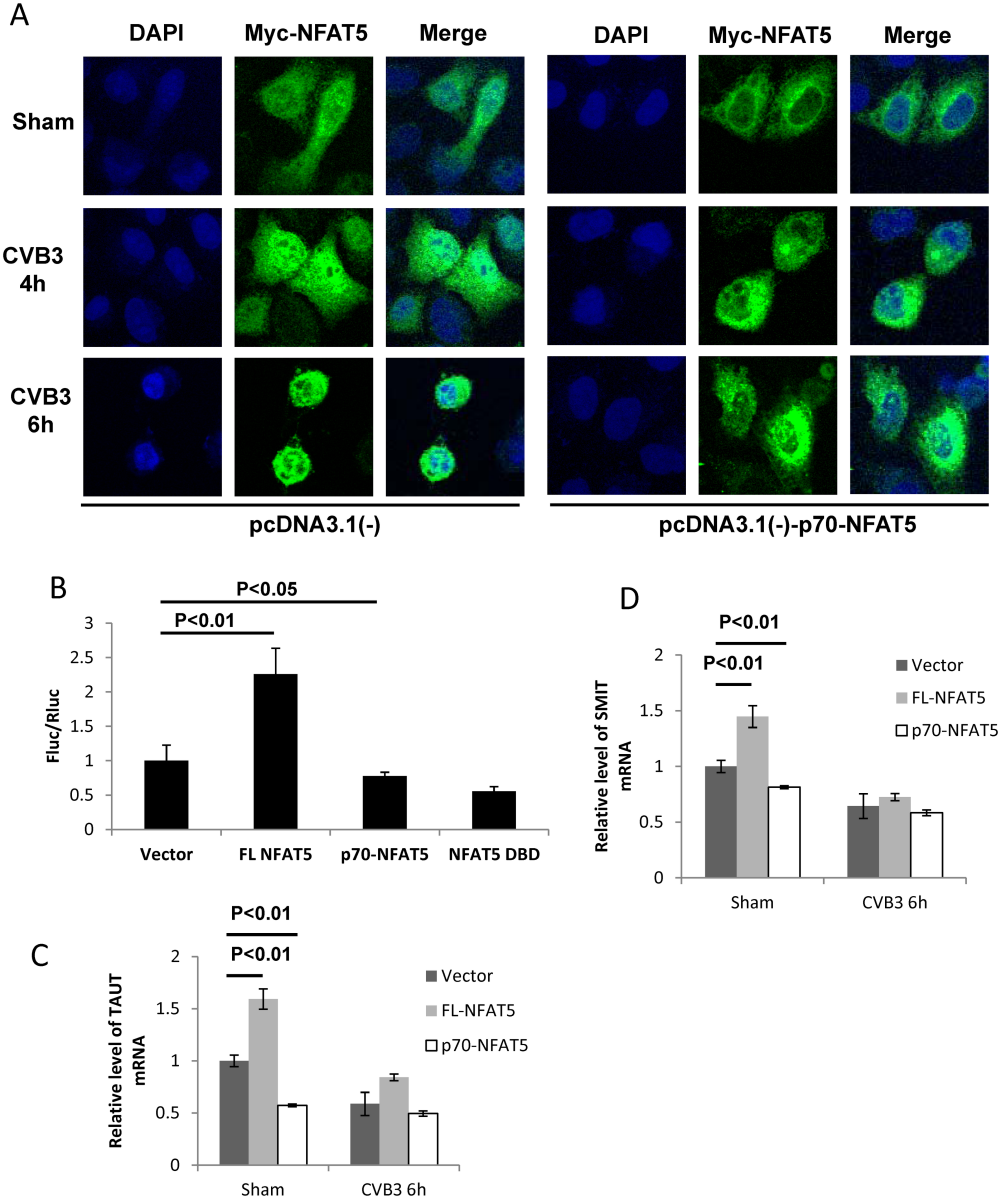
p70-NFAT5 acts as a dominant negative fragment of NFAT5. **(A)** HeLa cells were co-transfected with pEGFP-myc-NFAT5 and pcDNA3.1(-)-p70-NFAT5 or pcDNA3.1(-) empty vector. Then the cells were infected with CVB3. At 4 and 6 h pi, the cells were fixed and immunostained using a specific antibody against the myc tag and observed by confocal microscopy. **(B)** HeLa cells were co-transfected with the luciferase reporter constructs pGL-TonE-luciferase or pRL-polIII and the plasmids expressing FL NFAT5, p70-NFAT5, NFAT5 DBD or pEGFP empty vector. At 48 h pt, the cell lysates were subjected to dual luciferase assay detecting the activity of the firefly luciferase (Fluc) and the renilla luciferase (Rluc). The relative activity (FLu/RLu) was determined after normalization against the vector control. **(C, D)** HeLa cells were transfected with plasmids expressing FL NFAT5 or p70-NFAT5 and then infected with CVB3. At 6 h pi, the cellular RNAs were extracted for qPCR measurement of mRNA level of TauT **(C)** and SMIT **(D)** (normalized to GAPDH as described in Fig 1). Vector-transfected cells were used as a control. Three biological replicates were performed for each assay and the results was subjected to statistical analysis.

It has been reported that the N-terminal DNA-binding domain (aa 175–471) of NFAT5 (NFAT5 DBD) has a dominant negative effect on NFAT5 activity [27]. In our study, we first showed that the N-terminal cleavage product p70-NFAT5 demonstrated a positive effect on CVB3 replication to counteract the FL NFAT5 activity. We therefore speculated that p70-NFAT5 could be a dominant negative mutant of NFAT5. To verify this, we first determined the effect of p70-NFAT5 on the nuclear translocation of endogenous NFAT5, a key step of NFAT5 activation [29]. In cells transfected with pEGFP-myc-NFAT5, we observed a translocation of myc-NFAT5 from the cytoplasm to the nucleus (Fig 5A, **left panel**). This redistribution of FL NFAT5 after CVB3 infection was also supported by experiments using cells expressing myc-NFAT5^G503A^, the non-cleavable mutant of NFAT5, which showed similar translocation after CVB3 infection (S2 Fig). However, when p70-NFAT5 was expressed concurrently with myc-NFAT5, the redistribution of myc-NFAT5 was significantly decreased (Fig 5A, **right panel**). To further confirm the change of NFAT5 activity upon p70-NFAT5 overexpression, we cotransfected pGL-TonE-luciferase and the control luciferase plasmid pRL-polIII with plasmids expressing FL NFAT5, p70-NFAT5 or NFAT5 DBD into HeLa cells and then assayed for luciferase activity. The plasmid pGL-TonE-luciferase expressed firefly luciferase (Fluc) regulated by TonE, while the control plasmid pRL-polIII expressed Renilla luciferase (Rluc) regulated by a constitutively activated promoter, serving as an internal control. Thus, the ratio Fluc/Rluc indicated the activity of TonE. We found that FL NFAT5 enhanced luciferase expression, and therefore TonE activation, by more than 100% while p70-NFAT5 reduced it by ~25% and that the dominant negative effect of p70-NFAT5 was similar to that of NFAT5 DBD (Fig 5B). To further confirm NFAT5 activity on downstream genes upon p70-NFAT5 overexpression with or without CVB3 infection, we detected the mRNA levels of taurine transporter (TauT) [30] and sodium/myo-inositol transporter (SMIT), two genes directly regulated by NFAT5 [31, 32]. In sham-infected cells, we observed a ~60% increase of TauT mRNA when FL NFAT5 was overexpressed and a ~40% decrease when p70-NFAT5 was overexpressed (Fig 5C). As for SMIT mRNA, we observed a ~40% increase and a ~20% decrease when FL NFAT5 and p70-NFAT5 were overexpressed, respectively (Fig 5D). In CVB3-infected cells, we also observed the same trend of altered mRNA expression but the levels of differential expression were much lower than those in sham-infected cells (Fig 5C and 5D), probably due to the generation of p70-NFAT5 by 2A cleavage during infection. These results suggest the dominant negative effect of p70-NFAT5 on NFAT5 in both sham-infected and CVB3-infected cells.

### NFAT5 inhibits CVB3 replication in HeLa cells by inducing expression of iNOS

**Fig 6:**
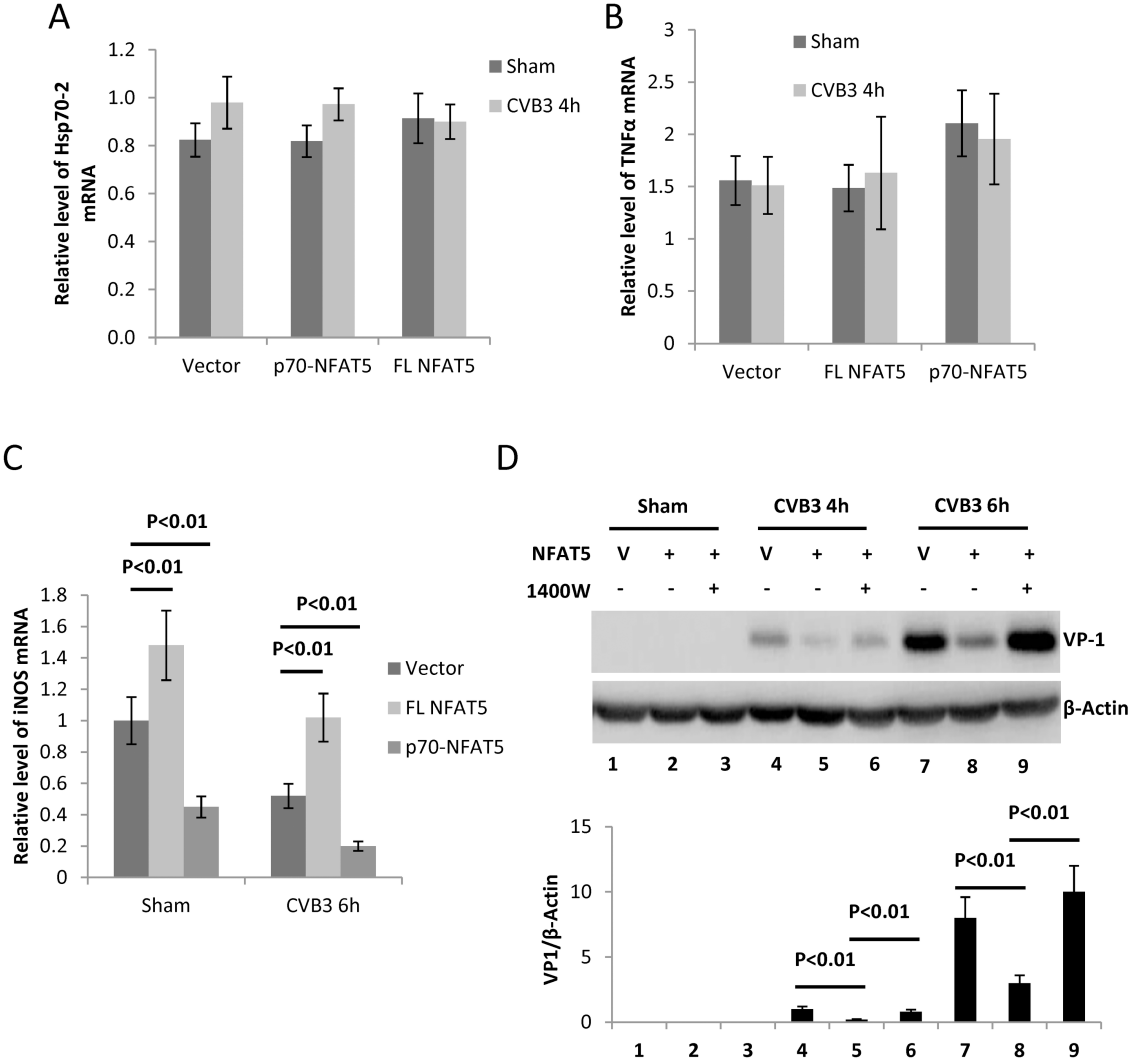
iNOS is essential for the anti-CVB3 effect of NFAT5. HeLa cells were transfected with plasmids expressing FL NFAT5 or p70-NFAT5 and then infected with CVB3. At 6 h pi, the cellular RNAs were extracted for qPCR measurement of mRNA levels of Hsp70-2 **(A)**, TNFα **(B)** and iNOS **(C)** (normalized to GAPDH as described in Fig 1). **(D)** HeLa cells transfected with pEGFP empty vector (V) or pEGFP-myc-NFAT5 were treated with 4 mM 1400W or DMSO control and then infected with CVB3 for VP1 detection and quantification (lower panel) as described in Fig 4A. Three biological replicates were performed for each assay and the result was subjected to statistical analysis.

Having uncovered the anti-CVB3 activity of NFAT5, we next aimed to reveal the underlying mechanism. Considering that NFAT5 is a transcription factor, we speculated that its anti-CVB3 activity might be attributed to the downstream genes regulated by NFAT5. Among these downstream genes of NFAT5, the molecular chaperone heat shock protein 70 (Hsp70) and transcription factor NFκB are reported to be related to CVB3 infection [33, 34]. Our results showed no significant changes (p>0.05) in Hsp70-2 expression or NFκB activity (indicated by TNFα expression, a marker of NFκB activation [19]) upon expression of FL NFAT5 or p70-NFAT5 in either sham-infected or CVB3-infected cells (Fig 6A and 6B). Nevertheless, the mRNA level of iNOS as assessed by qPCR, showed a ~40% increase upon FL NFAT5 overexpression and a ~50% decrease when p70-NFAT5 was overexpressed in both sham- and CVB3-infected cells (Fig 6C). It has been reported that nitric oxide generated by the catalysis of iNOS inactivates the Coxsackievirus protease 2A and 3C via S-nitrosylating the cysteine residue at the active site and thus interrupts the viral life cycle [22, 35]. Therefore, we speculated that NFAT5 inhibits CVB3 replication by inducing the expression of iNOS. To verify this speculation, we treated the NFAT5-overexpressing cells with 4 mM 1400W, a specific inhibitor of iNOS [36], and found that 1400W counteracted the effect of NFAT5 overexpression and restored the VP1 to a level even greater than that of the control (Fig 6D, **Lane 9 vs. 7**). These data indicate that iNOS induction is essential for the anti-CVB3 effect of NFAT5.

### Hypertonic treatment inhibits CVB3 replication in HeLa cells and promotes cell survival

**Fig 7:**
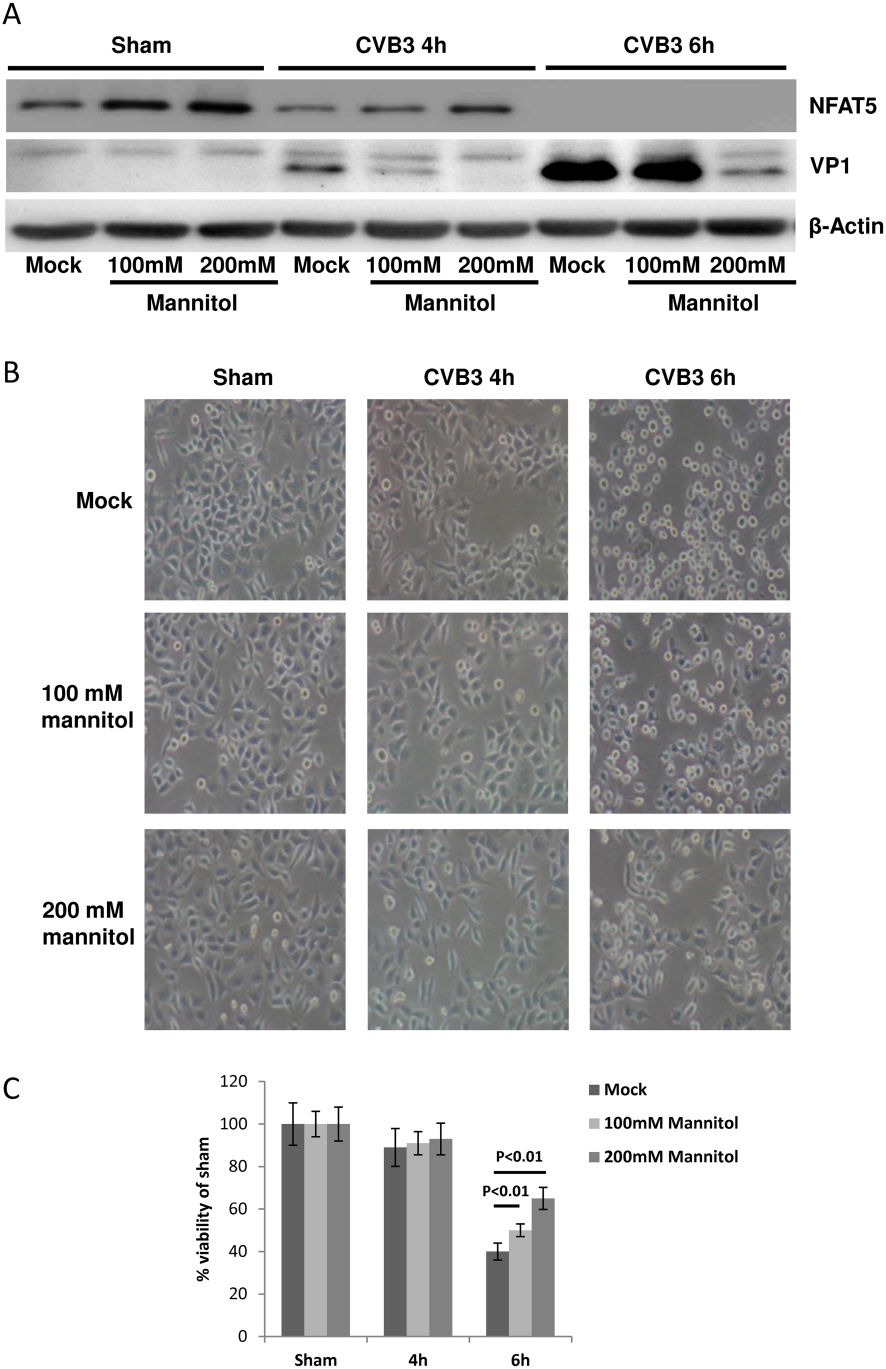
Hypertonic mannitol solutions inhibit CVB3 replication and promote cell survival during CVB3 infection. HeLa cells were treated with 100 mM, 200 mM mannitol solution or PBS and then subjected to CVB3 infection or sham-infection. At 4 and 6 h pi, cells were subjected to Western blot analysis of VP1 (**A**), phase contrast microscopy imaging (**B**) and MTS cell viability assay (**C**). The cell viability was determined by converting to the percentage of cell survival of the sham-infection control, which was set as 100%. Three biological replicates were performed for each assay and the result was subjected to statistical analysis.

The above experiments showed that a high level of NFAT5 is capable of inhibiting CVB3 replication. As a hypertonic responsive protein, NFAT5 can be induced by hypertonic stimulation, such as high concentration of sodium chloride (NaCl), which in turn may exert an anti-CVB3 effect. We therefore tested the effect of hypertonic NaCl solution on CVB3 replication. At 24 h before infection, we replaced the growth medium with Dulbecco’s modified Eagle’s medium (DMEM) containing an additional 100 mM NaCl, a concentration reported to be high enough to induce NFAT5 expression but that is not cytotoxic [37], and then infected the HeLa cells with CVB3. The hypertonic treatment resulted in an increase of NFAT5 expression (S3 Fig **Panel A, Lane 1 vs. 4**). At 4 and 6 h pi, a dramatic decrease of VP1 was observed in cells treated by HSS compared to the sham-treated control (S3 Fig **Panel A, Lanes 2–3 vs. Lanes 5-6**), suggesting that CVB3 replication is impaired by HSS treatment. The anti-CVB3 effect of HSS was further verified using cell viability assays. By using either imaging to assess cell morphology (S3 Fig **Panel B**) or an MTS cell viability assay (S3 Fig **Panel C**), we observed a significant increase in cell viability by ~30% at 6 h pi compared to the infected isotonic control. Considering that Na^+^ and Cl^-^ ions can pass the cell membrane via facilitated diffusion [38], which may exert unexpected effects on signaling pathways other than NFAT5, we employed mannitol, a non-permeable hyperosmotic agent [39], to validate the HSS experiments. Using different concentrations (0, 100 and 200 mM) of mannitol, we found that NFAT5 was increased and VP1 was decreased in a concentration-dependent manner and that the alterations of NFAT5 and VP1 expression were more significant (Fig 7A) with mannitol than with HSS treatment (S3 Fig **Panel A**). Furthermore, similar to HSS, hypertonic treatment with mannitol solutions of 100 or 200 mM also elevated the cell viability during CVB3 infection (Fig 7B and 7C). Altogether, our results suggest that hypertonic conditions inhibit CVB3 replication via NFAT5 expression.

### NFAT5 inhibits CVB3 replication in cardiomyocytes via iNOS

**Fig 8:**
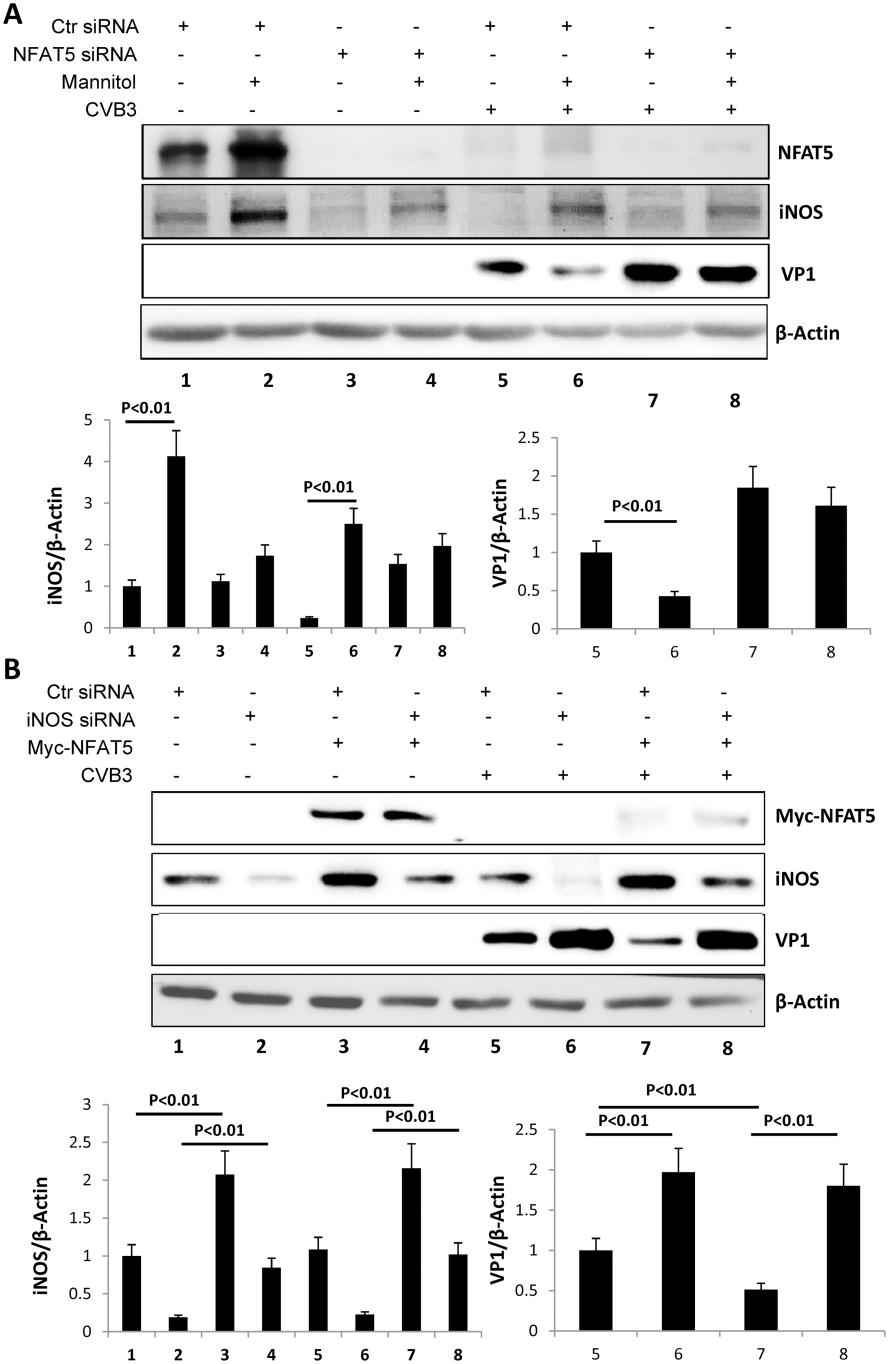
iNOS-mediated NFAT5 inhibition of CVB3 replication in HCM. (**A)** HCMs were transfected with NFAT5 siRNA or control (Ctr) siRNA. At 36 h pt, the cells were treated with 200 mM mannitol solution or PBS and then infected with CVB3 at an MOI of 40 or sham-infected with PBS. At 10 h pi, cells were subjected to Western blot analysis of NFAT5, iNOS and VP1. (**B**) HCMs were cotransfected with plasmids expressing myc-NFAT5 or empty vector pEGFP and iNOS siRNA or Ctr siRNA. At 36 h pt, the cells were infected with CVB3 at an MOI of 40 or sham-infected with PBS. At 10 h pi, cells were subjected to Western blot analysis of myc-NFAT5, iNOS and VP1. Quantification of iNOS and VP1 was performed as described in Fig 4A (lower panels). Three biological replicates were conducted for each assay and subjected to statistical analysis.

As CVB3 is a cardiotropic virus and a predominant pathogenic cause of myocarditis, it is important to understand the role of NFAT5 in CVB3-infected cardiomyocytes. To this aim, we overexpressed NFAT5 and NFAT5^G503A^ by plasmid transfection or knocked down NFAT5 using specific siRNAs in HCMs and then infected the cells with CVB3 at an MOI of 40. At 8 and 10 h pi, total protein was extracted from the cells and subjected to Western blot analysis of VP1. The overexpression of NFAT5 reduced the VP1 level at 8 h pi compared to vector-transfected cells and this reduction was more pronounced in NFAT5^G503A^-expressing cells (S4 Fig **Panel A**). On the other hand, NFAT5 knockdown enhanced VP1 expression compared to control siRNA-treated HCM (S4 Fig **Panel B**), which is similar to the results obtained using HeLa cells. Additionally, we treated sham- or CVB3-infected HCMs with 200 mM mannitol to induce NFAT5 expression and confirmed an increase of NFAT5 upon treatment (Fig 8A **Lane 1 vs. 2, Lane 5 vs. 6**). Simultaneously, iNOS level was significantly increased in cells treated with mannitol (**Lane 1 vs. 2, Lane 5 vs. 6**) and VP1 level was reduced by mannitol treatment (**Lane 5 vs. 6**). However, upon knockdown of NFAT5 by specific siRNAs, we observed a much weaker induction of iNOS expression (**Lane 2 vs. 4, Lane 6 vs. 8**) and impaired inhibition of VP1 in the mannitol treatment (**Lane 7 vs. 8**). These data indicate that hypertonic agents inhibit CVB3 replication in cardiomyocytes by inducing NFAT5. To further confirm that iNOS was involved in this pathway, we knocked down iNOS using specific siRNAs in NFAT5-overexpressing HCMs. We observed that VP1 level was dramatically increased upon iNOS knockdown (Fig 8B **Lane 5 vs. 6, Lane 7 vs. 8**) and that knockdown of iNOS did not affect NFAT5 level but rescued the inhibition of VP1 caused by NFAT5 overexpression (**Lanes 5 & 7 vs. 6 & 8**). These results confirmed that like in HeLa cells, in cardiomyocytes NFAT5 inhibits CVB3 replication via iNOS.

### Hypertonic saline lowers cardiac viral load and relieves tissue damage in mice

**Fig 9:**
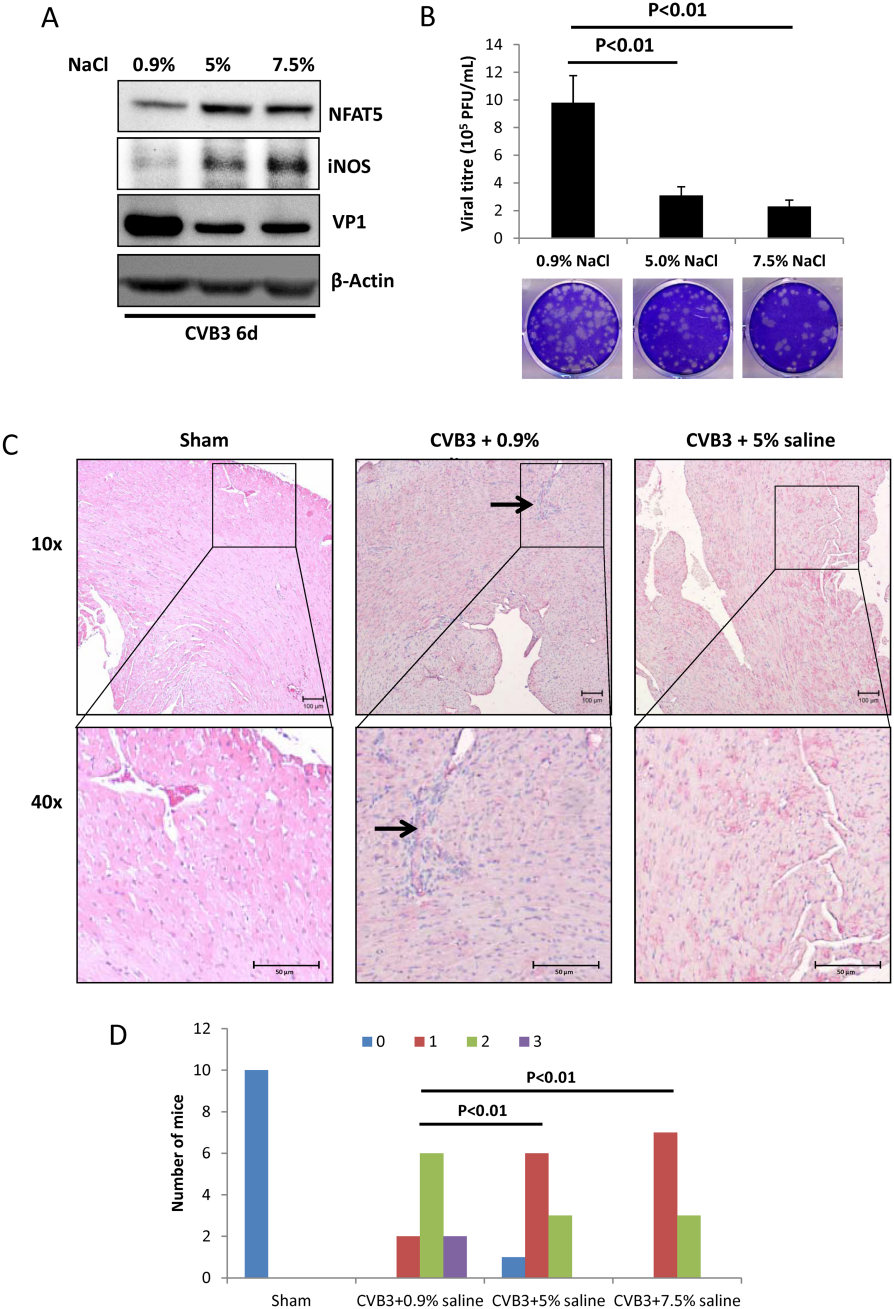
Hypertonic saline solution inhibits CVB3 replication and relieves mice heart tissue damage. Thirty 4-week-old A/J mice were randomly allocated equally into six groups and infected with CVB3 of 10^5^ pfu. NaCl solution at 0.9%, 5% and 7.5% concentration was IP injected into the mice daily during the infection, each concentration was administrated to two groups of mice (10 mice). At 6 d pi, the mice were euthanized and the heart tissues were harvested. Part of the heart tissue was homogenized for Western blot analysis of VP1 **(A)** and plaque assay to determine viral particle formation **(B)**. **(C)** The remaining heart tissues from these mice were fixed, sectioned and H&E stained to evaluate the tissue damage. The arrows indicated the inflammatory lesions with immune infiltration. Sham-infected /mock-treated mice were the additional controls. **(D)** The frequency of each myocarditis score in every treatment was shown in the histogram. The results were compared by χ^2^ test and p < 0.01 was considered as a significant difference.

To test whether the anti-CVB3 effect of HSS treatment occurs *in vivo*, 30 A/J mice were divided equally into three groups and intraperitoneally (IP) injected with 0.9% normal saline solution (NSS), and 5% and 7.5% HSS (10 mL/kg daily) for 6 consecutive days, respectively, and then the mice were challenged with CVB3 at 10^5^ pfu each. At 6 d pi, 3 of the 10 NSS-treated mice died and all HSS-treated mice survived. All remaining live mice were euthanized and heart tissue was collected for viral detection and histological analysis. Using immunoblotting and viral plaque assay of heart tissue, we found that the HSS-treated mice showed higher levels of NFAT5 and iNOS but lower levels of VP1 (Fig 9A) as well as decreased viral titre (Fig 9B) compared to the NSS-treated control mice, indicating that the viral load was reduced by HSS treatment. H&E staining of the myocardial tissue showed fewer lesions (the arrow in Fig 9C) with immune infiltration upon 5% HSS treatment compared to NSS treatment (Fig 9C). Note that since both 5% and 7.5% HSS showed a similar effect, both alleviating tissue injury, we only show the H&E staining for the 5% HSS treatment. The cardiac tissue damage of each mouse was further quantified by a blinded observer using the myocarditis scoring system reported previously [40] and the number of mice (frequency) with each myocarditis score was displayed as a histogram (Fig 9D). The histogram showed lower frequency of high myocarditis scores in HSS-treated groups compared to the NSS-treated group, and with a χ^2^ test, we showed a significant difference between HSS and NSS treatments, indicating less severe cardiac damage in HSS-treated groups, which further supports the imaging results. These data indicate that HSS is capable of relieving tissue damage during CVB3 infection by induction of NFAT5 expression.

## Discussion

Viral protease mediated cleavage of host proteins plays a critical role in viral replication and virus-induced tissue damage in picornavirus infections [41–45]. Therefore, identifying cleaved host proteins is a rational first step to discover potential drug targets in the development of anti-picornavirus therapeutics. NFAT5 is a transcription factor that is activated in hypertonic stress conditions. Thus, its activity can be modulated by changing the osmotic pressure of the extracellular fluid. However hypertonic stress occurs rarely in organs other than the kidney, thus few studies have linked NFAT5 to non-kidney diseases despite its ubiquitous expression throughout the body [14, 15]. Even though there is a lack of direct evidence, several previous reports provided clues as to NFAT5’s role in maintaining heart function. For instance, degradation of NFAT5 protein has a key role in doxorubicin-induced cytotoxicity in cardiac myocytes [16], NFAT5 is highly expressed in developing heart tissues [15] and it is critical for heart development in children and young adults [46]. However, the role of NFAT5 in infectious and inflammatory heart diseases has never been delineated. Thus, in this study, we used the myocarditis models of CVB3 infection of both a cardiomyocyte cell line and A/J mice to elucidate NFAT5’s role in CVB3 replication and CVB3-induced myocarditis.

First, we detected whether CVB3 infection would change NFAT5 level. It has been reported that NFAT5 expression is stimulated by p38 MAPK activation in response to hypertonic stress [47]. Considering that CVB3 infection activates p38 MAPK via phosphorylation [48], we were expecting to see NFAT5 upregulation and activation during CVB3 infection. Indeed, we observed a significant increase in NFAT5 protein level at early time points (~2 h pi) in HeLa cells infected with CVB3 (S5 Fig), but to our surprise, it decreased rapidly and was undetectable 4 h after infection. However, no NFAT5 mRNA decrease was observed during CVB3 infection, indicating that the reduction in NFAT5 protein level was not due to transcriptional arrest or mRNA degradation. We therefore switched our focus to the degradation and cleavage of NFAT5 protein during CVB3 infection. By using the inhibitors MG132 and z-VAD-fmk, we excluded the possibility of proteasome-mediated degradation and caspase-mediated cleavage of NFAT5, which are common causes of protein decrease during CVB3 infection [23, 49]. The most plausible reason remaining for NFAT5 reduction was cleavage mediated by viral protease 2A and/or 3C. Using an antibody against the N-terminal of NFAT5, we observed a ~70 kDa fragment (p70-NFAT5) after CVB3 infection. By using exogenous expression of 2A or 3C individually, we confirmed that both 2A and 3C were capable of cleaving NFAT5, but only 2A cleavage generated the 70 kDa N-terminal fragment p70-NFAT5, while 3C protease further cleaved the C-terminal fragment downstream of the 2A cleavage site. The NFAT5 functional domains are clustered in the N-terminal of NFAT5 (S1 Fig), including the nuclear export/localization signals (NES/NLS), auxiliary export domain (AED), DNA binding domain (RHD), etc. [25], and thus p70-NFAT5 is capable of translocating across the nuclear envelope and DNA binding. However, we found that the N-terminal fragment itself could not activate the expression of downstream genes, probably due to the lack of the C-terminal portion which harbors the transactivation domains of NFAT5 [27]. However, the N-terminal fragment competes with the intact NFAT5 for DNA binding, and can act as a dominant negative mutant of NFAT5 [27]. Although the 2A cleavage product p70-NFAT5 (aa 1–502) is different from the previously reported dominant negative mutant (aa 175–471), our results show that p70-NFAT5 can still inhibit the activity of endogenous NFAT5.

The next issue we addressed was the mechanism underlying the anti-CVB3 effect of NFAT5. It is known that CVB3 RNA transcription relies on its RNA-dependent RNA polymerase and its translation employs an IRES-driven mechanism. Apparently, the transcription factor NFAT5 does not directly affect CVB3 multiplication; instead, it may indirectly promote expression of downstream target genes involved in the antiviral signal cascade. The direct downstream genes regulated by NFAT5 include organic osmolyte-related genes [30–32, 50, 51], molecular chaperone genes [37], and iNOS genes [52]. The expression of the transcription factor NFκB is not regulated by NFAT5, but NFAT5 binding modulates NFκB activity [19], thus, NFκB can also be considered a downstream effector of NFAT5. Among these downstream proteins, the organic osmolyte-related genes, such as TauT and SMIT, are mainly involved in osmotic regulation, and no antiviral effects or cardiac functions of these genes have been reported. In a previous study, we showed that the chaperone protein Hsp70 promotes CVB3 replication by stabilizing the CVB3 genomic RNA [33], but in this current study, NFAT5 overexpression did not significantly change Hsp70-2 expression, an Hsp70 gene regulated by NFAT5 [37]. Similar to Hsp70-2 expression, no significant change in NFκB activity was observed when NFAT5 was overexpressed, even though NFκB was reported to be activated during CVB3 infection [21]. However, iNOS expression was upregulated by the overexpression of FL NFAT5 and downregulated by the overexpression of p70-NFAT5 in either sham- or CVB3-infected cells. These data suggested that iNOS was a potential candidate for a mechanistic link between NFAT5 and CVB3 replication. The expression of iNOS is induced by a toll-like receptor (TLR), which requires NFAT5 activity [52]. CVB3 infection activates TLR expression [53], but high level of iNOS catalyzes production of nitric oxide, which impairs CVB3 replication via inhibition of viral proteases [22, 35]. Thus, we hypothesized that CVB3 might cleave and inactivate NFAT5 to block the induction of iNOS. This hypothesis was supported by our experiment showing that treatment of cells with iNOS inhibitor 1400W or iNOS siRNA restored CVB3 replication that was blocked by the overexpression of NFAT5 (Fig 6D).

The anti-CVB3 activity of NFAT5 makes it a potential drug target during CVB3 infection. As a hypertonicity-responsive protein, NFAT5 can easily be induced by hypertonic solutions. HSS, which contains a high concentration of NaCl, is a good choice for induction of NFAT5 due to its easy accessibility. In our experiments, HSS treatment significantly reduced viral replication and enhanced cell viability in CVB3-infected cells. In our *in vivo* test, A/J mice treated with HSS showed a lower viral load in heart tissue compared to NSS-treated controls. Furthermore, histological evaluation showed a lower level of heart tissue damage and immune infiltration in HSS-treated mice, indicating that HSS treatment reduced the severity of CVB3-induced myocarditis. Although HSS can effectively induce NFAT5, NaCl is an ionic osmolyte capable of entering cells and increasing intracellular ion concentration [38]. Excess intracellular ions may lead to increased reactive oxygen species formation, cytoskeletal rearrangements, increased mitochondrial depolarization, and decreased DNA replication and DNA repair [54, 55]. To exclude these potential side effects of HSS, we repeated these experiments inducing NFAT5 expression with mannitol, a non-permeable organic hyperosmolyte, and determined its effect on CVB3 replication *in vitro*. Interestingly, mannitol showed a similar anti-CVB3 effect to HSS, decreasing viral replication and enhancing cell survival during CVB3 infection.

In summary, our study uncovers a novel function of the transcription factor NFAT5 in cardiac protection during CVB3 infection. CVB3 multiplication depends on the appropriate processing of its polyprotein by viral proteases 2A and 3C to complete its life cycle [7, 8]. However, the activity of viral proteases is inhibited by NFAT5 via enhanced expression of its downstream target protein iNOS, which catalyzes production of the viral protease inhibitor NO. Since FL NFAT5 protein level is decreased due to cleavage of the protein by viral protease during infection, upregulating NFAT5 expression is a rational strategy to counteract CVB3 infection. Our discovery of the anti-CVB3 activity of the two hypertonic agents HSS and mannitol may be the first exploratory step in the development of low-cost and easily accessible compounds to treat infectious heart disease. Although exciting, this study on the therapeutic potential of hypertonic agents is quite preliminary and far from clinical trial. Potential adverse side effects are a major concern for the clinical application of HSS. Thus search for other hypertonic agents to induce upregulation of NFAT5 may be a strategy for the development of effective therapy against viral myocarditis.

## Materials and methods

### Ethics statement

This study was carried out in strict accordance with the recommendations in the Guide to the Care and Use of Experimental Animals—Canadian Council on Animal Care. All mouse experiments were performed according to the animal experiment protocols approved by the Animal Care Committee of Faculty of Medicine, University of British Columbia (protocol number: A16-0093). Mice were infected by IP injection with 10^5^ pfu of CVB3 or sham-infected with PBS. During the infection period, the mice were treated by IP injection with 0.9%, 5% or 7.5% NaCl solution in a dose of 10 mL/kg daily. Heart tissues were collected at day 6 pi after euthanasia in CO2, fixed in 10% buffered formalin, embedded in paraffin and subjected to H&E staining.

### Cell culture, animals, viral infection and treatment

HeLa cells (ATCC) were cultured in DMEM supplemented with 100 μg/ml penicillin-streptomycin, 2 mM L-glutamine and 10% fetal bovine serum (FBS) (Sigma). SV40 immortalized HCM were purchased from Applied Biological Materials (ABM, Richmond, BC, Canada) and cultured in Prigrow I medium (ABM) with 100 μg/ml penicillin-streptomycin (Thermo Fisher), 2 mM glutamine (Thermo Fisher) and 10% fetal FBS. Hypertonic medium was made by adding NaCl or mannitol directly to DMEM to make the final concentrations indicated in the experiments.

CVB3 (CG) strain was a kind gift from Dr. Charles Gauntt (University of Texas Health Science Center) and propagated in HeLa cells. Virus stock was prepared from the cells by three freeze-thaw cycles followed by centrifugation to remove cell debris and stored at -80°C. The titer of virus stock was determined by plaque assay as described below. Cell cultures were infected with CVB3 at an MOI of 10 for 1 h (HeLa) or at an MOI of 40 for 1.5 h (SV40 cardiomyocytes) in a serum-free medium, washed with PBS (Thermo Fisher), and then replenished with fresh medium containing FBS. Male A/J mice (4-week old) were purchased from the Jackson Laboratory. Mice were infected by IP injection with 10^5^ pfu of CVB3 or sham-infected with PBS. During the infection period, the mice were treated by IP injection with 0.9%, 5% or 7.5% NaCl solution in a dose of 10 mL/kg daily. Heart tissues were collected at day 6 pi after euthanasia in CO_2_, fixed in 10% buffered formalin, embedded in paraffin and subjected to H&E staining as described previously [56].

### Constructs, molecular cloning and mutagenesis

pEGFP-myc-NFAT5 and pEGFP-myc-NFAT5-DBD were kind gifts from Dr. Anjana Rao (Addgene plasmid #13627 and #14112) [57]. The FLAG-p70-NFAT5 fragment was amplified by PCR using universe hot start high-fidelity DNA polymerase per manufacturer’s instructions and pEGFP-myc-NFAT5 as the template (Bimake). The primers (NFAT5 F/Xho I and NFAT5 G503 R/Bam HI) used in this PCR are listed in S1 Table. The PCR product was digested with Xho I and Bam HI and then inserted into pcDNA3.1(-) to generate the expression plasmid of p70-NFAT5, pcDNA3.1(-)-p70-NFAT5.

The plasmid pEGFP-myc-NFAT5 was mutated at G503 or G650 by PCR-mediated site-directed mutagenesis using corresponding primers (S1 Table) to generate pEGFP-myc-NFAT5^G503A^ and pEGFP-myc-NFAT5^G650A^, respectively. Briefly, PCR reactions using mutagenesis primers and pEGFP-myc-NFAT5 template were conducted using the same DNA polymerase kit as described above, and then the PCR-producted mutant plasmid was transformed into DH5α competent *E*. *coli* cells. The transformed cells were plated onto Luria broth (LB) plates containing 50 μg/mL kanamycin and incubated overnight at 37°C. The following day, individual bacterial colonies were picked and plasmid was extracted using High-Speed Plasmid Mini Kit (Froggabio). The mutation sites were verified by DNA sequencing using primers NFAT5 G503 Seq and NFAT5 G650 Seq (S1 Table).

### Transfection of plasmids and siRNAs

All of the siRNAs were purchased from Santa Cruz Biotechnology and transfected into cells using Oligofectamine (Life Technologies) according to the manufacturer’s instructions. Briefly, 2 × 10^5^ HeLa cells or HCM were grown at 37°C overnight to 30–40% confluence in 6-well plates, washed with PBS and overlaid for 6 h with transfection complex containing siRNAs and Oligofectamine. The transfection medium was then replaced with DMEM containing 10% FBS and the incubation was continued for 48 h for HeLa cells and 36 h for HCM. The plasmids were transfected using the same procedures as those described for siRNAs except that Lipofectamine 2000 (Life Technologies) was used as transfection reagent and the initial cell confluence was 80–90%. The following analyses were performed at 24 h or 36 h pt.

### RNA extraction and quantitative real-time PCR

Total cellular RNA was extracted using RNeasy mini kit (Qiagen) according to the manufacturer’s instructions. cDNAs were then synthesized by reverse transcription using SuperScript III First-Strand Synthesis System (Invitrogen) and detected by qPCR using QuantiTect SYBR Green PCR kit (Qiagen). The mRNA level of glyceraldehyde 3-phosphate dehydrogenase (GAPDH) was detected and used as the endogenous control to normalize the data. All qPCR experiments were performed in triplicates with no template as a negative control. The primers for the qPCR are showed in S1 Table.

### Western blot analysis

Cells were washed with cold PBS before the addition of an appropriate volume of RIPA lysis buffer (Santa Cruz). After incubation for 20 min on ice, the cell lysates were centrifuged at 13,000 × g for 15 min at 4°C, and protein-containing supernatant was collected. The isolated proteins were separated by 10% SDS-PAGE and transferred onto nitrocellulose membranes. Membranes were blocked with 5% skim milk in Tris-buffered saline with 0.5% Tween 20 (TBST) and incubated overnight with one of the following primary antibodies: monoclonal rabbit anti-VP1, monoclonal rabbit anti-FLAG and monoclonal rabbit anti-myc (Thermo Fisher); monoclonal mouse anti-β-actin and polyclonal rabbit anti-NFAT5 (Santa Cruz). After several washes with TBST, each blot was further incubated with an appropriate secondary antibody (goat anti-mouse or donkey anti-rabbit IgG) conjugated to horseradish peroxidase (Thermo Fisher). Detection was carried out by enhanced chemiluminescence (Amersham) as per the manufacturer’s instructions. β-actin was detected as a loading control. Signal intensities were quantified by using the ImageJ (NIH) program and normalized to the control samples (set as 1.00).

### Cell viability assay

Cellular morphology was observed and photographed at room temperature under a phase contrast microscope (TMS-F, Nikon) connected with a camera (Coolpix 8400, Nikon). Cell viability was further quantified using a 3-(4-5-dimethylthiazol-2-yl)-5- (-3-carboxymethoxyphenyl)-2H-tetrazolium salt (MTS) assay kit following the manufacturer’s instructions (Promega). Briefly, cells were incubated with MTS solution for 2 h. Absorbency of formazan was measured at 492 nm using enzyme-linked immunosorbent assay (ELISA) plate reader.

### Viral plaque assay

Samples of cells and medium from plates receiving the various treatments were freeze-thawed and then centrifuged (4,000 × g) to collect the supernatant containing virus. HeLa cells were seeded onto 6-well plates (8 × 10^5^ cells/well) and incubated at 37°C for 20 h to reach a confluence of approximately 90% and then washed with PBS and overlaid with 800 μl of virus-containing samples serially diluted in cell culture medium. After a viral adsorption period of 60 min at 37°C, the supernatant was removed and the cells overlaid with 2 ml of sterilized soft Bacto-agar-minimal essential medium, cultured at 37°C for 72 h, fixed with Carnoy’s fixative for 30 min, and stained with 1% crystal violet. The plaques were counted and viral pfu per ml was calculated.

### Reporter construction and dual luciferase assay

The NFAT5 reporter plasmid pGL-TonE-luciferase and the control Renilla reporter plasmid pRL-PolIII were kind gifts from Dr. Anjana Rao (Addgene plasmid # 14110) [57] and Dr. Norbert Perrimon (Addgene plasmid # 37380) [58], respectively. In the pGL-TonE-luciferase plasmid, the sequence containing TonE from the promoter region of the human aldose reductase gene, a typical downstream gene of NFAT5 [50], is inserted upstream of the firefly luciferase coding region, and thus the expression of firefly luciferase is regulated by NFAT5 activity [57]. We cotransfected pGL-TonE-luciferase with FL NFAT5 or p70-NFAT5 into HeLa cells. At 48 h pt, the cell lysates were used for luciferase assay on a Tecan GENios fluorescence reader to determine the relative luciferase activity (Firefly/Renilla) by using the Dual-Luciferase Reporter Assay System (Promega) as per the manufacturer’s instructions. Each treatment was done in three biological replicates.

### Immunofluorescence and confocal microscopy

Cells cultured on glass cover slips (Thermo Fisher) were washed with PBS, fixed with 4% paraformaldehyde, and permeabilized with methanol/acetone (1:1) for 20 min at −20°C. Cells were then washed with TBS (25 mM Tris, 0.15M NaCl, pH 7.2 to 7.5) twice and blocked with 2.5% BSA in TBS for 1 h at room temperature followed by incubation overnight at 4°C with polyclonal rabbit anti-NFAT5 antibody (Santa Cruz) diluted in blocking buffer. Cells were then washed with TBS five times at room temperature. Slides were stained with goat anti-rabbit IgG (H + L) labeled with Alexa Fluor 488 and then incubated for 1 h at room temperature. After a final wash, the slides were stained with DAPI (DAKO) and mounted with nail polish. Images were captured using a Leica AOBS SP2 confocal microscope (Leica, Allendale, NJ) as described previously [33].

### Scoring of myocarditis

Scoring of myocarditis in the cardiac tissue of mice was conducted using a 0–4 scoring system [40]. Briefly, on the H&E stained sections, normal muscle cells were stained pink, while nuclei and other basophilic structures were stained blue-purple. Inflammatory lesions with immune infiltration were seen as a clustering of nucleated cells with an associated necrosis. The severity of myocarditis was evaluated by the following scoring system: Score 0 indicated lack of myocarditis; Score 1 indicated very limited focal distribution of myocardial lesions; Score 2 described multiple lesions; Score 3 described multiple lesions with some confluent and extensive necrosis; and Score 4 described the presence of coalescent and pervasive lesions spreading throughout most of the observed tissue. The frequency of each myocarditis score was displayed as a histogram and subjected to χ^2^ test. We did not observe Score 4 myocarditis in our experiments, thus we did not include Score 4 in our histogram.

### *In vitro* cleavage assay

Cleavage reactions were performed in 20 mM Hepes (pH 7.4), 150 mM potassium acetate and 1 mM DTT buffer. Purified recombinant CVB3 2A and 3C proteases were kind gifts from Dr. Eric Jan of the University of British Columbia [59]. Mouse heart homogenate was mixed with 2A^pro^ at 5 ng/μl or 3C^pro^ at 100 ng/μl. Reaction mixtures were incubated at 37°C for 8 h and then the reaction was stopped by the addition of SDS-PAGE sample buffer. Cleavage activity was assessed by Western blot analysis.

### Statistical analysis

The Student’s *t* test was employed to analyze the data. The results are expressed as means ± standard deviations (SD) of at least three independent experiments. A *p* value less than 0.05 was considered statistically significant.

## Supporting information

S1 Fig Protease 2A cleavage site in the NFAT5 protein. REL-homology domain (RHD) contains the DNA-binding domain of NFAT5. The region upstream of RHD contains a canonical nuclear export signal (NES), a consensus bipartite nuclear localization signal (NLS) and an auxiliary export domain (AED). The large arrow indicates the 2A cleavage site at amino acid G503. Protease cleavage produced a ~70 kDa N-terminal fragment, p70-NFAT5.
(TIFF)

S2 Fig NFAT5^G503A^ was translocated from the cytoplasm to the nucleus during CVB3 infection. HeLa cells were transfected with pEGFP-myc-NFAT5^G503A^ and then infected with CVB3 at an MOI of 10. At 4 and 6 h pi, the cells were fixed and immunostained using a specific antibody against the myc tag and observed by confocal microscopy.
(TIFF)

S3 Fig Hypertonic saline solution inhibits CVB3 replication and promotes cell survival during CVB3 infection. HeLa cells were pre-treated with additional 100 mM NaCl or isotonic medium for 4 h and then infected with CVB3. At 4 and 6 h pi, the cells were subjected to Western blot analysis of VP1 protein **(A)**, phase contrast morphological imaging **(B)** and MTS cell viability assay **(C)**. Cell viability was determined by converting to the percentage of cell survival of the sham-treatment control, which was set as 100%.
(TIFF)

S4 Fig NFAT5 negatively regulates CVB3 replication in SV40 human cardiomyoctes. HCMs were transfected with plasmids expressing myc-NFAT5 or myc-NFAT5G503A mutant. At 24 h pt, the cells were infected with CVB3 at an MOI of 40 or sham-infected with PBS. At 10 h pi, the cells were subjected to Western blot analysis of myc-NFAT5 and VP1 (A) and plaque assay detecting viral progeny release (B). Another batch of HCMs were transfected with NFAT5 siRNA or control siRNA. At 36 h pt, the cells were infected with CVB3 and subjected to Wstern blot (C) and plaque assay (D) as described above.
(TIFF)

S5 Fig NFAT5 is upregulated at early stages of CVB3 infection. HeLa cells were infected with CVB3 at an MOI of 10 or sham-infected with PBS. At the indicated time points post infection, cell lysates were used for Western blot analysis of NFAT5 protein using an antibody against the N-terminal epitope of NFAT5. β-actin was used as a loading control.
(TIFF)

S1 Table Primers used in the study. (PDF)
