# Supplementary figures and images for: Cleavage of osmosensitive transcriptional factor NFAT5 by Coxsackieviral protease 2A promotes viral replication
Source: PLoS Pathog. 2017 Dec 8;13(12):e1006744. doi: 10.1371/journal.ppat.1006744 (PMC5738146; doi:10.1371/journal.ppat.1006744)

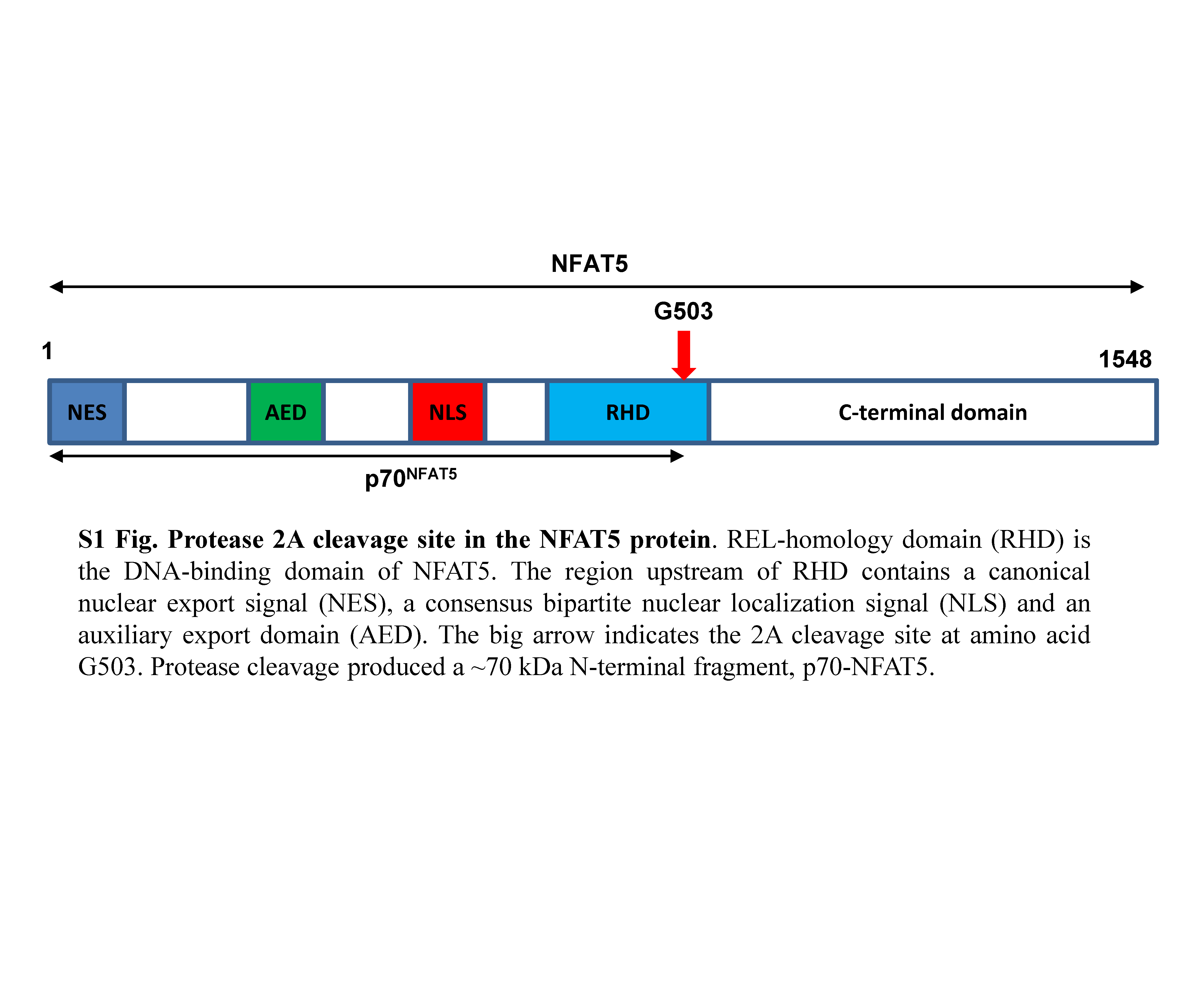

Supplement: S1 Fig — REL-homology domain (RHD) contains the DNA-binding domain of NFAT5. The region upstream of RHD contains a canonical nuclear export signal (NES), a consensus bipartite nuclear localization signal (NLS) and an auxiliary export domain (AED). The large arrow indicates the 2A cleavage site at amino acid G503. Protease cleavage produced a ~70 kDa N-terminal fragment, p70-NFAT5. (TIFF) [file ppat.1006744.s001.tiff]

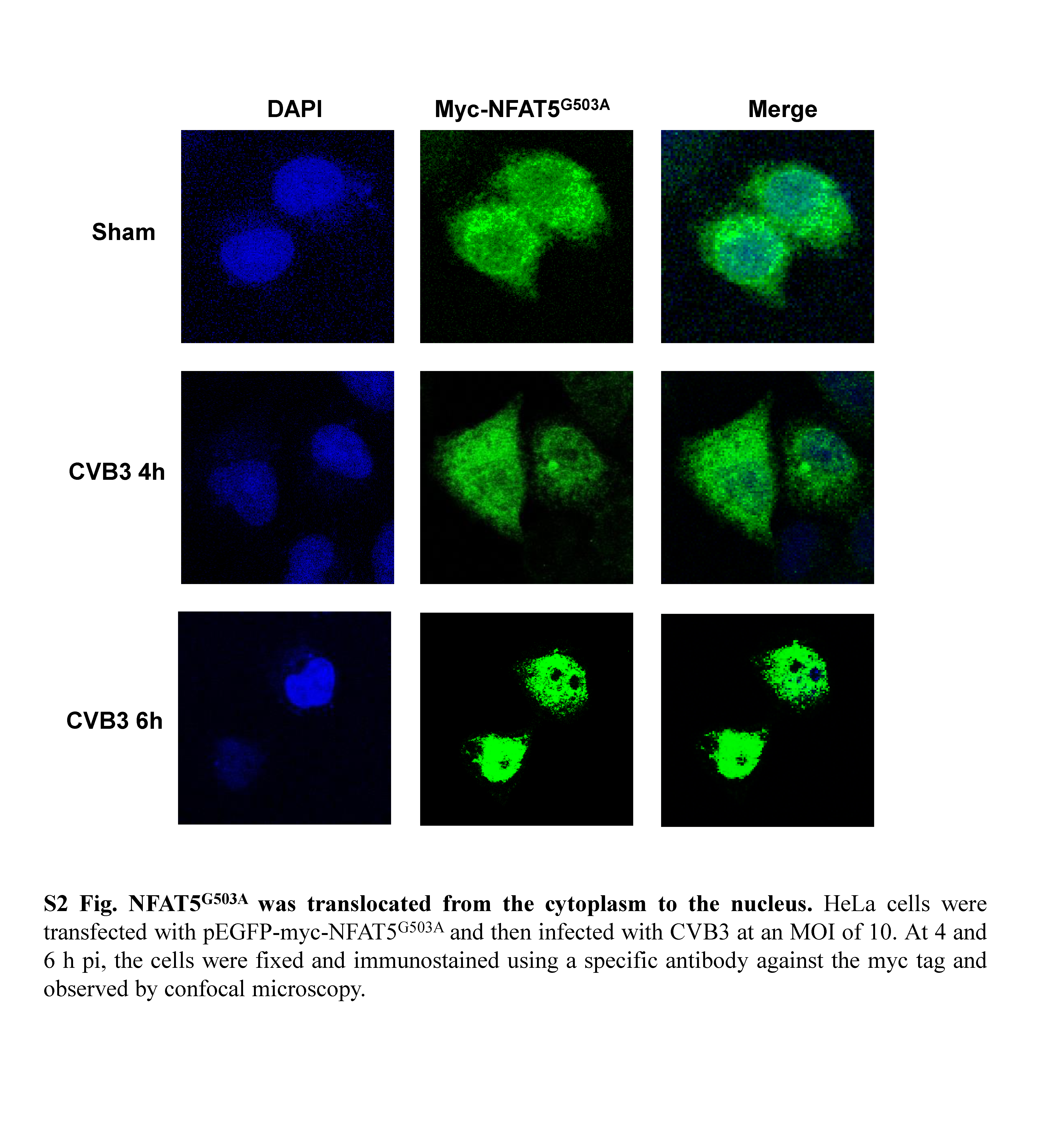

Supplement: S2 Fig — HeLa cells were transfected with pEGFP-myc-NFAT5G503A and then infected with CVB3 at an MOI of 10. At 4 and 6 h pi, the cells were fixed and immunostained using a specific antibody against the myc tag and observed by confocal microscopy. (TIFF) [file ppat.1006744.s002.tiff]

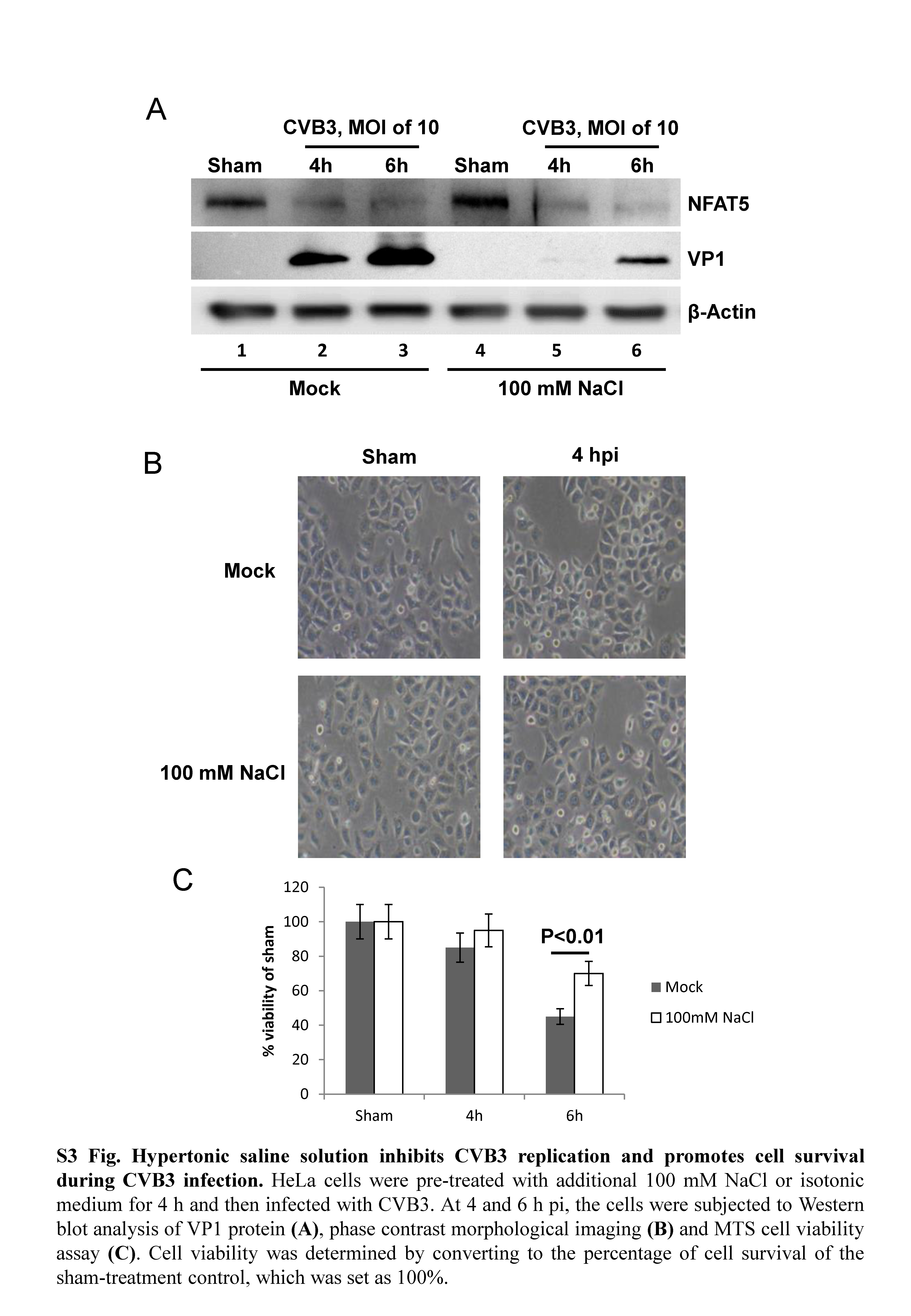

Supplement: S3 Fig — HeLa cells were pre-treated with additional 100 mM NaCl or isotonic medium for 4 h and then infected with CVB3. At 4 and 6 h pi, the cells were subjected to Western blot analysis of VP1 protein (A), phase contrast morphological imaging (B) and MTS cell viability assay (C). Cell viability was determined by converting to the percentage of cell survival of the sham-treatment control, which was set as 100%. (TIFF) [file ppat.1006744.s003.tiff]

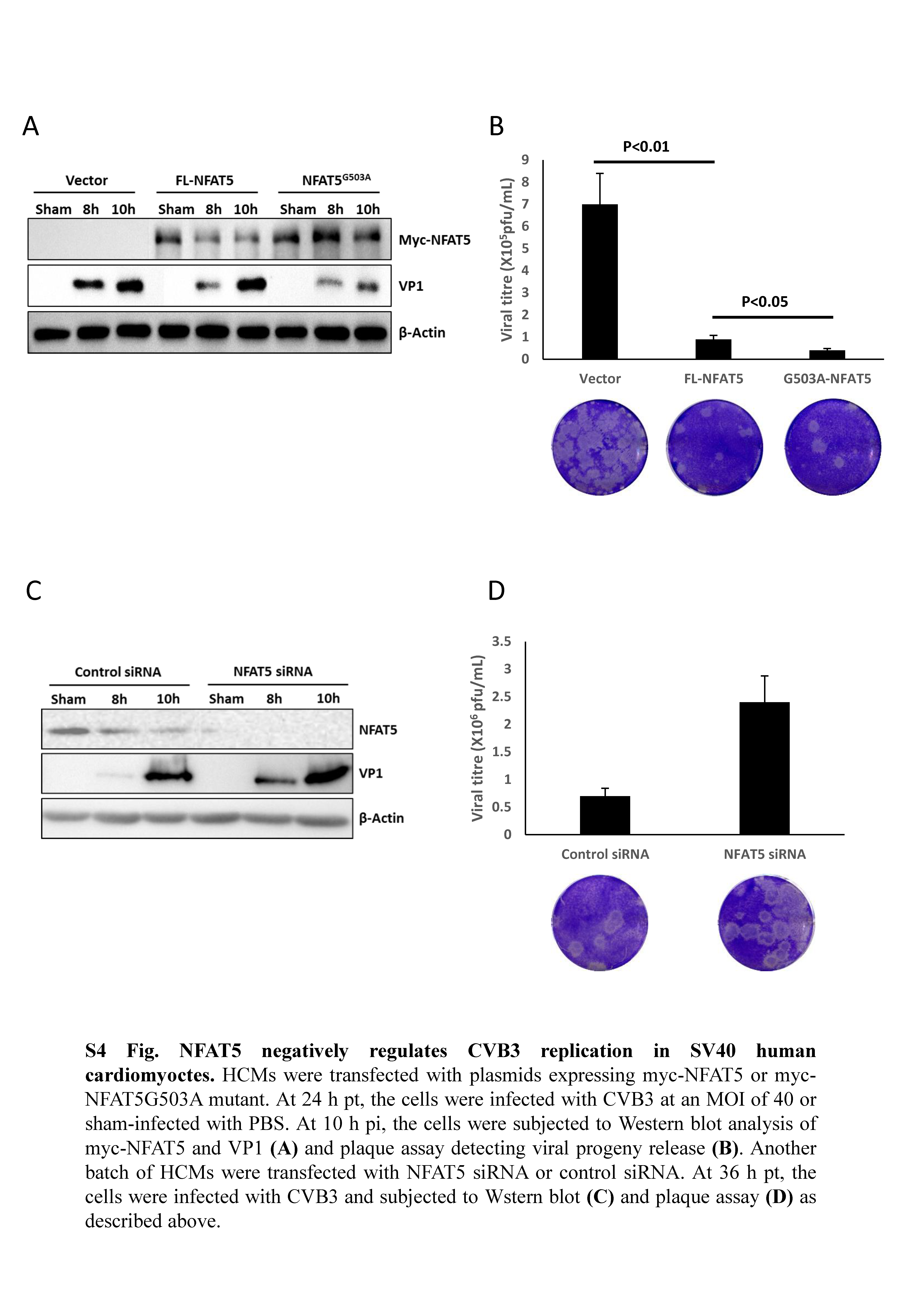

Supplement: S4 Fig — HCMs were transfected with plasmids expressing myc-NFAT5 or myc-NFAT5G503A mutant. At 24 h pt, the cells were infected with CVB3 at an MOI of 40 or sham-infected with PBS. At 10 h pi, the cells were subjected to Western blot analysis of myc-NFAT5 and VP1 (A) and plaque assay detecting viral progeny release (B). Another batch of HCMs were transfected with NFAT5 siRNA or control siRNA. At 36 h pt, the cells were infected with CVB3 and subjected to Wstern blot (C) and plaque assay (D) as described above. (TIFF) [file ppat.1006744.s004.tiff]

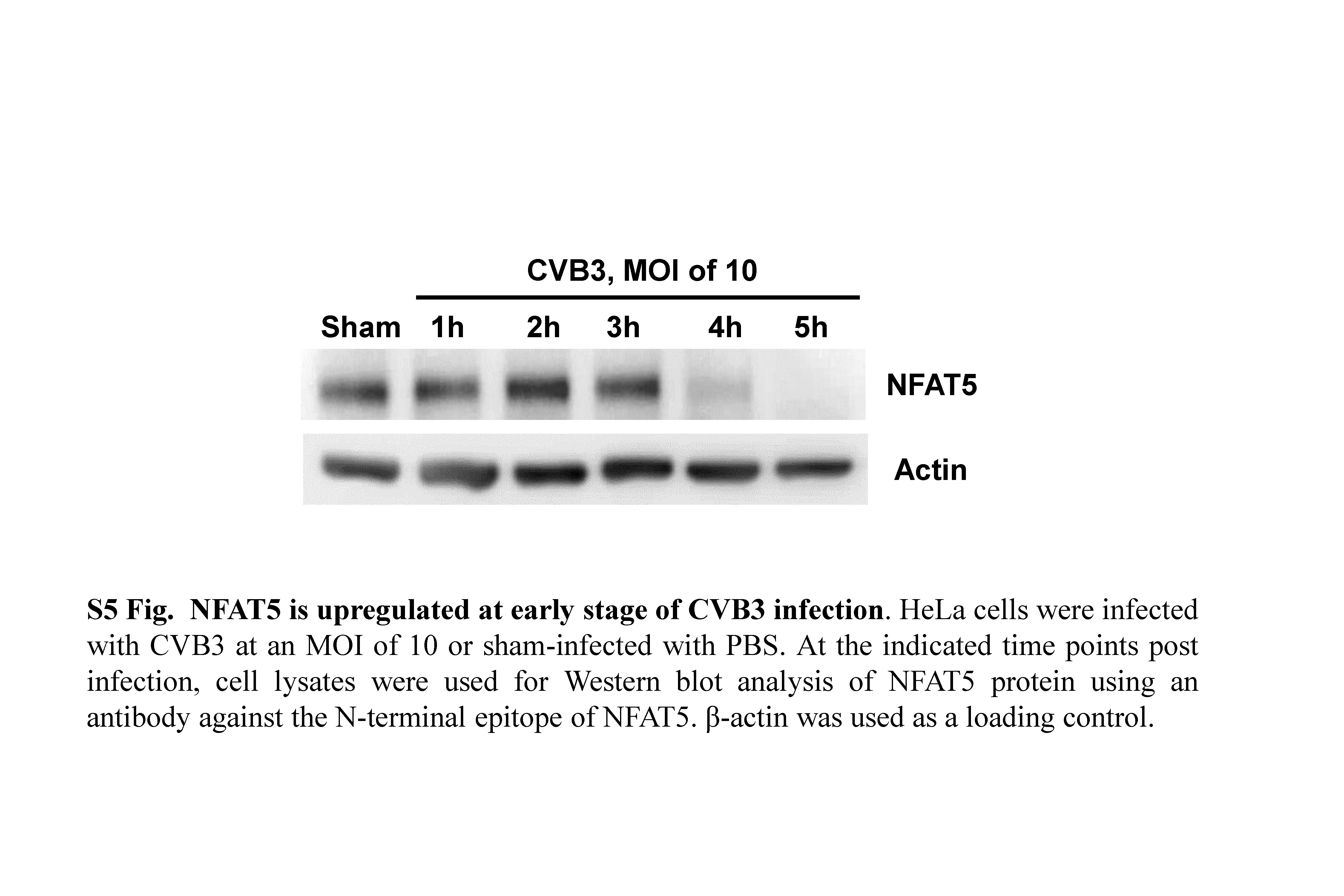

Supplement: S5 Fig — HeLa cells were infected with CVB3 at an MOI of 10 or sham-infected with PBS. At the indicated time points post infection, cell lysates were used for Western blot analysis of NFAT5 protein using an antibody against the N-terminal epitope of NFAT5. β-actin was used as a loading control. (TIFF) [file ppat.1006744.s005.tiff]
