# Supplementary material for: Cleavage of osmosensitive transcriptional factor NFAT5 by Coxsackieviral protease 2A promotes viral replication
Source: PLoS Pathog. 2017 Dec 8;13(12):e1006744. doi: 10.1371/journal.ppat.1006744 (PMC5738146; doi:10.1371/journal.ppat.1006744)
Supplement: S1 Table — (PDF) [file ppat.1006744.s006.pdf]

Table S1. Primers used in the study

Experiment	Primer	Sequence
Q-RT-PCR	Human Hsp70-2 forward	TGTTTGTCTTTGAGGTGGAC
	Human Hsp70-2 reverse	AAGAATTCTAATGAACATATCGGTTG
	Human NFAT5 forward	GAAGTGGACATTGAAGGCACT
	Human NFAT5 reverse	CTGGCTTCGACATCAGCATT
	Human TauT forward	AGATCATCATAGGCCAGTACAC
	Human TauT reverse	TAGACATTCAGGAGGGACACA
	Human iκB forward	GATCCGCCAGGTGAAGGG
	Human iκB reverse	GCAATTTCTGGCTGGTTGG
	Human iNOS forward	GCAGAATGTGACCATCATGG
	Human iNOS reverse	ACAACCTTGGTGTTGAAGGC
	Human GAPDH forward	AATCCCATCACCATCTTCCA
	Human GAPDH reverse	TGGACTCCACGACGTACTCA
	Mouse GAPDH forward	GGCAAATTCAACGGCACAGT
	Mouse GAPDH reverse	AGATGGTGATGGGCTTCCC
	CVB3 2A forward	ATCAAGTTGCGTGCTGTG
	CVB3 2A reverse	TGCGAAATGAAAGGAGTGT
Molecular cloning	NFAT5 F/XhoI	AGCTCTCGAGATGGGCGGTGCTTGCAGCTCC
	NFAT5 G503 R/BamHI	AGCTGGATCCTTACTTATCGTCGTCATCCTTGTAATCAGTAGTTTTCATTGCTTTC
	NFAT5 G503A F	GGCCATGAAAGCAATGAAAACTACTGCATGTAATTTAGATAAGGTAAATATTATCCC
	NFAT5 G503A R	GGGATAATATTTACCTTATCTAAATTACATGCAGTAGTTTTCATTGCTTTCATGGCC
	NFAT5 G650A F	GATATTACAGTCAGATGCTACAGTGGTTAATTTGTCAC
	NFAT5 G650A R	GTGACAAATTAACCACTGTAGCATCTGACTGTAATATC
	NFAT5 G503 Seq	ACTTACACTCCAGACCC
	NFAT5 G650 Seq	TGACAACTATTCAAACCC
